# A molecular systems architecture of the mesenchymal stromal cell microenvironment

**DOI:** 10.1093/stmcls/sxaf042

**Published:** 2025-08-22

**Authors:** V A Shiva Ayyadurai, Prabhakar Deonikar, Vishvatha Radhakrishnan, Armand Keating

**Affiliations:** Systems Biology Group, CytoSolve Research Division, CytoSolve, Inc., Cambridge, MA 02138, United States; Open Science Institute, International Center for Integrative Systems, Cambridge, MA 02138, United States; Systems Biology Group, CytoSolve Research Division, CytoSolve, Inc., Cambridge, MA 02138, United States; Open Science Institute, International Center for Integrative Systems, Cambridge, MA 02138, United States; Systems Biology Group, CytoSolve Research Division, CytoSolve, Inc., Cambridge, MA 02138, United States; Open Science Institute, International Center for Integrative Systems, Cambridge, MA 02138, United States; University Health Network, Toronto, ON M5G 2C4, Canada; University of Toronto, Toronto, ON M5S 1A1, Canada

**Keywords:** mesenchymal stromal cells, CytoSolve, systems biology, molecular systems architecture, immunomodulation, tissue regeneration, fibrosis

## Abstract

A systems-level understanding of immunomodulatory, regenerative, and pro-/antifibrosis functions of mesenchymal stromal cells (MSCs) is critical to advance MSCs as a viable therapeutic option. Given the complexity of MSCs and their interactions with microenvironmental cells, a systems biology approach may enable such understanding to achieve practical objectives such as target identification, combination therapeutics, clinical strategies, and avoidance of adverse effects. In this study, a molecular systems architecture of MSCs microenvironment is developed to organize the complexity of biomolecular interactions between MSCs and other microenvironmental cells. This architecture provides a visual mapping of MSC interactions, identifies the complex crosstalk between MSCs and cells in the microenvironment, reveals potential targets, and offers a framework for creating future predictive, quantitative computational (*in silico*) models of the MSC microenvironment. The development of combination therapeutics, clinical strategies to improve therapeutic efficacy, and avoidance of adverse effects can be facilitated by such *in silico* models. However, it must all begin with a molecular systems architecture of MSCs—the objective and result of this study.

## Graphical Abstract

**Graphical Abstract:**
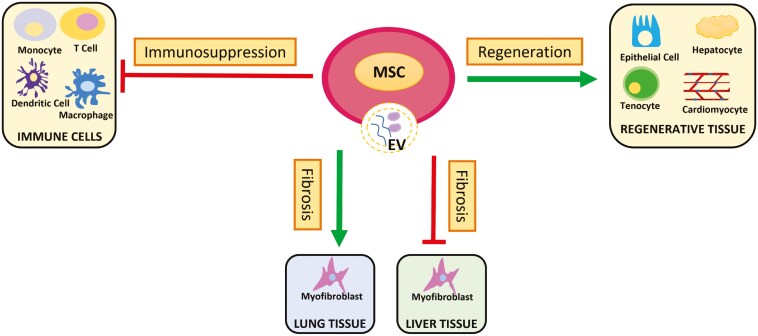

**Significance Statement**

Systems biology approaches hold great promise for advancing a systems-level understanding of the complex interactions of mesenchymal stromal cells (MSCs) and the microenvironment that lead to immunosuppression, regeneration, and pro-/antifibrosis. A molecular systems architecture is developed that visually maps the complexity of MSCs’ interactions with the microenvironment. This architecture demonstrates its viability for applications by identifying molecular targets of MSCs. In the future, such an architecture can enable the construction of predictive, quantitative computational models to uncover combination therapies, clinical solutions, and avoidance of adverse effects.

## Introduction

Therapeutic application of mesenchymal stromal cells (MSCs) has gained increasing momentum owing to their immunomodulatory ability, potential to differentiate into multiple cell types, and ability to modulate fibrotic activity in several tissues,^1-3^ which is evidenced by over 9000 studies conducted over the past 30 years.^4^ These studies have predominantly relied on a necessary but reductionist approach to understand how MSCs interact with their microenvironment to elicit therapeutic effects. One of the key objectives of this study is to employ systems biology methods to advance clinical applications of MSCs. The development of a *molecular systems architecture* of the MSC microenvironment aims to: (1) aid in the visualization of the MSC microenvironment that comprises complex biochemical interactions within its cellular systems; (2) reveal the biological processes and the underlying molecular pathways implicated in the therapeutic potential of MSCs; (3) identify molecular species that may be targeted by MSCs for therapeutic applications; and (4) provide a blueprint of molecular interactions that can be used to develop quantitative and predictive mathematical models that may lead to combination therapeutics, clinical strategies to improve therapeutic efficacy, and the avoidance of adverse effects.

Systems biology provides a framework to understand biological functions in living organisms, which result from dynamic networks of biochemical interactions. A dysfunction in one or more of the signaling cascades in any of these networks leads to either a functional gain or loss leading to the pathogenesis of a disease. The majority of treatments for these diseases are derived from a reductionist approach that tends to have significant weaknesses, such as the inability to develop combination therapies, “one-size-fits-all” solutions that do not account for heterogeneity in the patient population, and the inability to utilize novel computational frameworks that can assist in understanding systems-level off-target effects that lead to adverse side effects.^5-7^ A systems biology approach, specifically the development of a molecular systems architecture, is hypothesized to address these weaknesses.

A systems biology methodology is developed in this study to conduct a systematic literature review of the present understanding of MSCs and their immunomodulatory, regenerative, and pro/antifibrotic effects but also to develop a molecular systems representation, namely an interactome, of the molecular interactions between MSCs and microenvironmental cells. These insights may provide the MSC research field with a detailed molecular systems map to understand the complex biochemical reactions between MSCs and their microenvironment. The outcomes of this investigation may enable future research efforts in identification of interventions using MSCs.

Network systems of molecular pathways span multiple organ systems, tissues, cells, and cellular compartments in a complex disease or a biological process. In previous works by the authors, a systems biology approach was employed to develop molecular systems architectures—network systems of signaling cascades of molecular interactions—for complex diseases. This approach resulted in the identification, aggregation, and integration of molecular pathway networks in cellular microenvironments for C1 metabolism in plants,^8^ neurovascular disease,^9^ low-grade chronic inflammation,^10^ periodontal disease,^6^ liver detoxification,^11^ acute myeloid leukemia (AML),^7^ and amyotrophic lateral sclerosis (ALS).^12^ These molecular systems architectures have been employed to develop predictive quantitative computational models to support in vitro, in vivo, and clinical research.^13,14^

Mesenchymal stromal cells are characterized by their elongated, multipotent nature and are distributed throughout the mesenchymal stroma or connective tissues across the body.^15,16^ Mesenchymal stromal cells are precursors for diverse cell types,^17^ namely adipocytes, chondrocytes, and osteoblasts.^18,19^ These cells are essential for maintaining and repairing tissue health and balance, operating through multiple communication strategies including autocrine and paracrine signaling via the release of extracellular vesicles (EVs) containing secretomes^20^ and direct cell-to-cell contact.^21^ These secretomes contain soluble factors such as growth factors,^22^ immunomodulatory cytokines,^23,24^ active proteins, lipids, messenger RNAs (mRNAs), long noncoding RNAs, or microRNAs (miR).^25^

Mesenchymal stromal cells are derived from various sources such as amniotic membrane, umbilical cord,^26^ intra-pancreatic tissue,^27^ gingival MSCs,^28^ menstrual blood-derived MSCs,^20^ Wharton’s jelly,^29^ and human tonsils.^30^ Unstimulated MSCs derived from various sources generally exhibit a fibroblast-like spindle morphology.^31-33^ Plasticity of MSCs is their ability to alter their phenotype and functions in response to their microenvironment.^34^ Interactions with neighboring cells, protease exposure, and hypoxia—along with intracellular factors like microRNA expression—significantly influence MSC function and fate across tissues.^35^ The characteristic surface biomarkers present on the unstimulated MSCs based on their origin are listed in Table S3.

Each type of MSC exhibits differences in their immunomodulatory properties. The supernatants from umbilical cord MSCs (UC-MSCs) had a higher level of monocyte chemoattractant protein-1 (MCP-1), transforming growth factor (TGF)-β1, platelet-derived growth factor D (PDGFD), and prostaglandin E2 (PGE2).^21^ Amniotic membrane MSCs (AM-MSCs) exhibit higher PGE2 expression and indoleamine 2,3-dioxygenase (IDO) activity compared with UC-MSCs when primed by interferon-γ (IFN-γ) and/or tumor necrosis factor (TNF)-α induction.^26^

Mesenchymal stromal cells actively modulate the innate immune system through specific mechanisms, including the inhibition of neutrophil infiltration, oxidative burst, and extracellular trap release, as well as the reduction of natural killer (NK) cell activation and cytotoxicity.^36^ Furthermore, MSC activation in an inflammatory environment leads to the adoption of an anti-inflammatory phenotype.^37-39^ The immunomodulatory nature of MSCs makes them potential candidates in treating graft-versus-host disease^40^ as well as cellular therapies targeting inflammatory and autoimmune disorders, including type 1 diabetes (T1D).^39^

**Figure 1.**
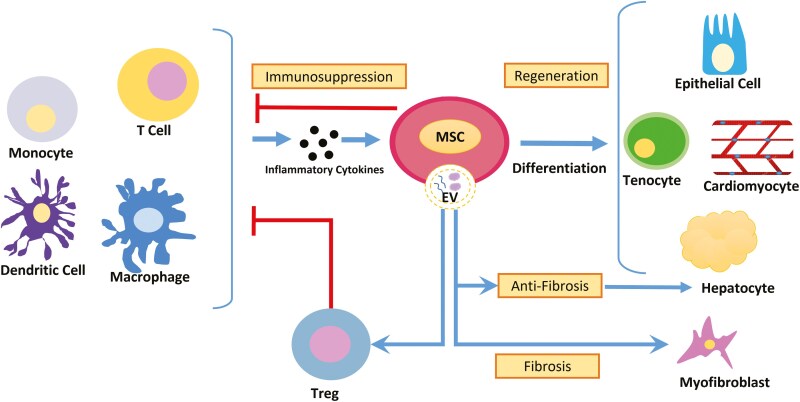
The MSC microenvironment. Monocytes, DCs, PBMCs, macrophages, and T cells through inflammatory cytokines lead to MSCs’ immunosuppression. MSCs differentiate into tenocytes, cardiomyocytes, hepatocytes, and epithelial cells leading to tissue regeneration. MSCs-derived secretory factors released in the EVs promote differentiation into myofibroblasts leading to fibrosis and activation of Tregs leading to immunosuppression.

Mesenchymal stromal cells enhance wound healing and tissue repair cell growth through paracrine cytokine signaling. Mesenchymal stromal cells promote cell migration, proliferation, and activation in human dermal fibroblasts^41^ and also play a role in angiogenesis, constricting collagen deposition, and accelerating wound closure.^42^ Transplantation of MSC significantly reduced metabolic dysfunction and enhanced liver regeneration by increasing the hepatocytes and sinusoidal endothelial cells proliferation, and lowering fat buildup in hepatocyte.^43^ In the lungs, MSCs promote tissue repair by upregulating vascular remodeling and cytoskeletal remodeling.^44^ Mesenchymal stromal cells can also play a therapeutic role in promoting hair growth,^45^ chondrogenesis,^46^ and meniscus repair.^47^ The MSCs interact with other cells in various microenvironments, leading to immunosuppression, tissue regeneration, and fibrosis, as shown in Figure 1.

## Methods

The scientific literature is searched to identify journal papers that contain research on MSCs, molecular interactions between MSCs and other cells in the microenvironment that are involved in immunomodulation, regeneration, and fibrosis. CytoSolve—a systems biology tool employed in this study—aids in systematic bioinformatics literature review and building computational (*in silico*) models of biological pathways.^10,48-51^ Applications of CytoSolve include diverse areas of research, including ALS,^12^ osteoarthritis,^52^ AML,^7^ neurovascular diseases,^9^ and periodontitis,^6^ to name a few. The protocol for setting up and using CytoSolve is provided in Supplementary Information.

### Literature review process

The critical step in building a molecular systems architecture is to find and curate peer-reviewed journal articles from public databases. CytoSolve^48^ is used to conduct the systematic bioinformatics literature review process that allows for screening, archival, distributed collaborative review, and curation of the pertinent literature. The following steps were involved in the literature review process:

1. Generate a list of keywords that include Medical Subject Headings (MeSH) to improve extraction and relevance of peer-reviewed journal articles
2. Search and extract the pertinent peer-reviewed journal articles published between January 2018 and December 2023 from public databases, including Google Scholar, Medline, and PubMed, to create an “Initial Set”
3. Shortlist the most relevant articles based on their titles and abstracts from the Initial Set repository to create the “Final Set”
4. Conduct a comprehensive review of peer-reviewed journal articles in the Final Set

### Inclusion criteria

A comprehensive review of the article text is performed by the authors. Relevance of an article is determined based on whether the MeSH keywords with specific relation to MSCs in Table S2 appear in the article. The article search restricted the studies to include data from humans in vivo or in vitro experiments. Data from unpublished literature were not considered due to lack of peer review. MeSH keywords are listed in Table S2.

The screening, eligibility, and finalization process for identifying and shortlisting articles for the literature review is documented in PRISMA diagram (Figure S2).^53^

## Results

The CytoSolve systematic bioinformatics analysis resulted in the molecular systems architecture of the MSC microenvironment represented by (1) a schematic representing a multilayered molecular systems architecture, (2) an interactome of molecular pathways representing the molecular systems architecture, and (3) interactome of biomolecular interactions in subsystems of the molecular systems architecture.

### Four-layered molecular systems architecture schematics

**Figure 2.**
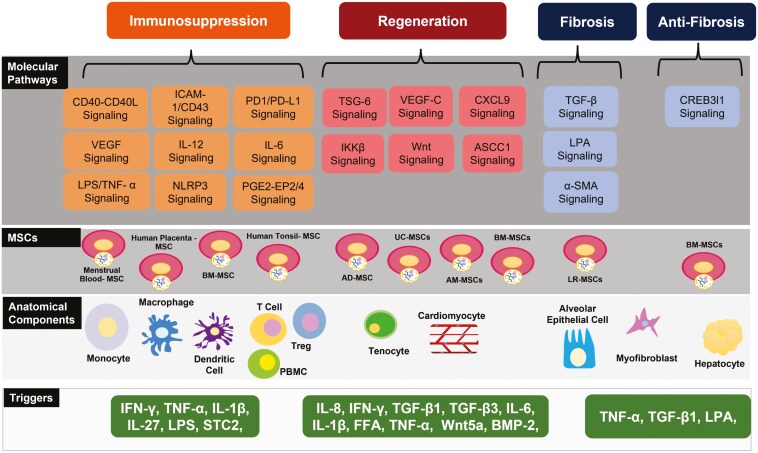
Molecular systems architecture of MSCs. In the 4-layered architecture, the bottom layer, Triggers, consists of potential triggers: Inflammatory cytokines and other factors that are implicated in affecting the physiology and functions of MSCs. The second layer from the bottom, Anatomical Components, consists of the anatomical components of the MSC microenvironment and the cells MSCs interact with either through paracrine, autocrine signaling or cell-to-cell contact: NK cells, DC, macrophage, T cell, myofibroblasts, chondrocytes, epithelial cells, adipocytes, and cardiomyocytes. The third layer from the bottom, MSCs layer, consists of different types of MSCs. The fourth layer from the bottom, molecular pathways, consists of the molecular pathways within and among the anatomical components. The top layer reveals the 4 biological processes that the MSCs are involved in: immunosuppression, regeneration, fibrosis, and antifibrosis.

The 4 layers of MSC molecular systems architecture are shown in Figure 2 and described in detail below.

#### The triggers layer

The Trigger layer consists of inflammatory cytokines. The most common forms of immunosuppression exhibited by MSCs include T cell suppression, increased proliferation of Treg cells, and skewing of macrophages to the M2 phenotype, an anti-inflammatory phenotype. Apart from immunosuppression, MSCs are also involved in regeneration that involves cell differentiation, proliferation, and angiogenesis.

Exposure of MSCs to pro-inflammatory cytokines such as interleukin (IL)-1β, TNF-α, and IFN-γ leads to increased expression of immunosuppressive species such as soluble factors, immunomodulatory proteins (eg, IDO, PGE2), and miRs from the MSCs.^38,54^ The MSC secretome also contains vascular endothelial growth factor (VEGF) that plays a major role in angiogenesis and wound healing.^42^ Mesenchymal stromal cells play both fibrotic and antifibrotic roles. In the lungs, MSCs differentiate into myofibroblasts and enhance fibrosis in the lungs,^15^ whereas they are found to inhibit fibrosis in the liver.^55^

#### The anatomical components layer

The second layer from the bottom, as shown in Figure 2, represents cellular components of the various cells that interact with MSCs: NK cells, dendritic cell (DC), macrophages, T cells, myofibroblasts, chondrocytes, epithelial cells, adipocytes, and cardiomyocytes. Interactions among these cell types occur across various molecular pathways that are implicated in MSCs function.

#### The MSCs layer

The third from the bottom represents different MSCs that interact with the microenvironmental cells. The types of MSCs represented in this study include AM-MSCs, adipose-derived MSCs (AD-MSCs), UC-MSCs, bone marrow-derived MSCs (BM-MSCs), gingival MSCs, menstrual blood-derived MSCs, Wharton’s jelly-derived MSCs, and human tonsil-derived MSCs (TMSCs). Mesenchymal stromal cells are involved in autocrine and paracrine signaling via the release of EVs containing secretomes. These secretomes contain soluble factors such as growth factors, immunomodulatory cytokines, active proteins, lipids, mRNAs, long noncoding RNAs, or miRs.

#### The molecular pathways layer

Seventeen molecular pathways that represent the crosstalk across microenvironmental cells and MSCs are represented in the fourth layer from the bottom, as shown in Figure 2. These molecular pathways lead to biological processes of MSCs. These 17 pathways are: cluster of differentiation (CD)40-CD40-L signaling, intercellular adhesion molecule (ICAM)-1/CD-43 signaling, programmed death (PD)1/PD-L1 signaling, VEGF signaling, IL-12 signaling, IL-6 Signaling, lipopolysaccharide (LPS) and TNF-α signaling, PGE2—E-prostanoid (EP) 2/4 signaling, nucleotide-binding oligomerization domain-like receptor pyrin domain containing 3 (NLRP3) signaling, TNF-stimulated gene (TSG)-6 signaling, VEGF-C signaling, chemokine (C-X-C motif) ligand (CXCL)9 signaling, inhibitor of nuclear factor-κB (IκB) kinase (IKK) β signaling, Wnt signaling, activating signal cointegrator 1 complex subunit 1 (ASCC1) signaling, TGF-β signaling, lysophosphatidic acid (LPA) signaling, and α-smooth muscle actin (α-SMA) signaling.

These molecular pathways lead to 4 critical biological processes controlled by MSC: immunosuppression, regeneration, fibrosis, and antifibrosis. Immunosuppression is affected by CD40-CD40L signaling, ICAM-1/CD-43 signaling, PD-1/PD-L1 signaling, VEGF signaling, IL-12 signaling, IL-6 signaling, LPS and TNF-α signaling, PGE2-EP2/4 signaling, and NLRP3 signaling. Regeneration is affected by TSG-6 signaling, VEGF-C signaling, CXCL9 signaling, IKKβ signaling, Wnt signaling, and ASCC1 signaling. In hepatocytes, BM-MSCs promote cyclic adenosine monophosphate responsive element binding protein 3 like 1 (Creb3l1), leading to antifibrosis effects, whereas in lungs, lung resident MSCs (LR-MSCs) promote fibrosis via activation of TGF-β, LPA signaling, and α-SMA signaling.

### Molecular systems architecture representing the interactome of cellular biochemical pathways

**Figure 3.**
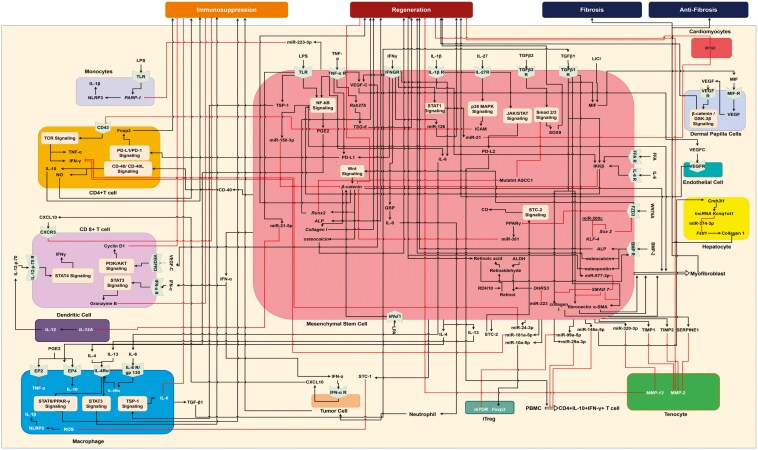
Interactome of the molecular systems architecture of the MSC microenvironment. Molecular pathways interactions are represented across the MSCs and multiple cell types, including NK cells, DC, macrophage, T cell, myofibroblasts, chondrocytes, epithelial cells, adipocytes, and cardiomyocytes.

**Figure 4.**
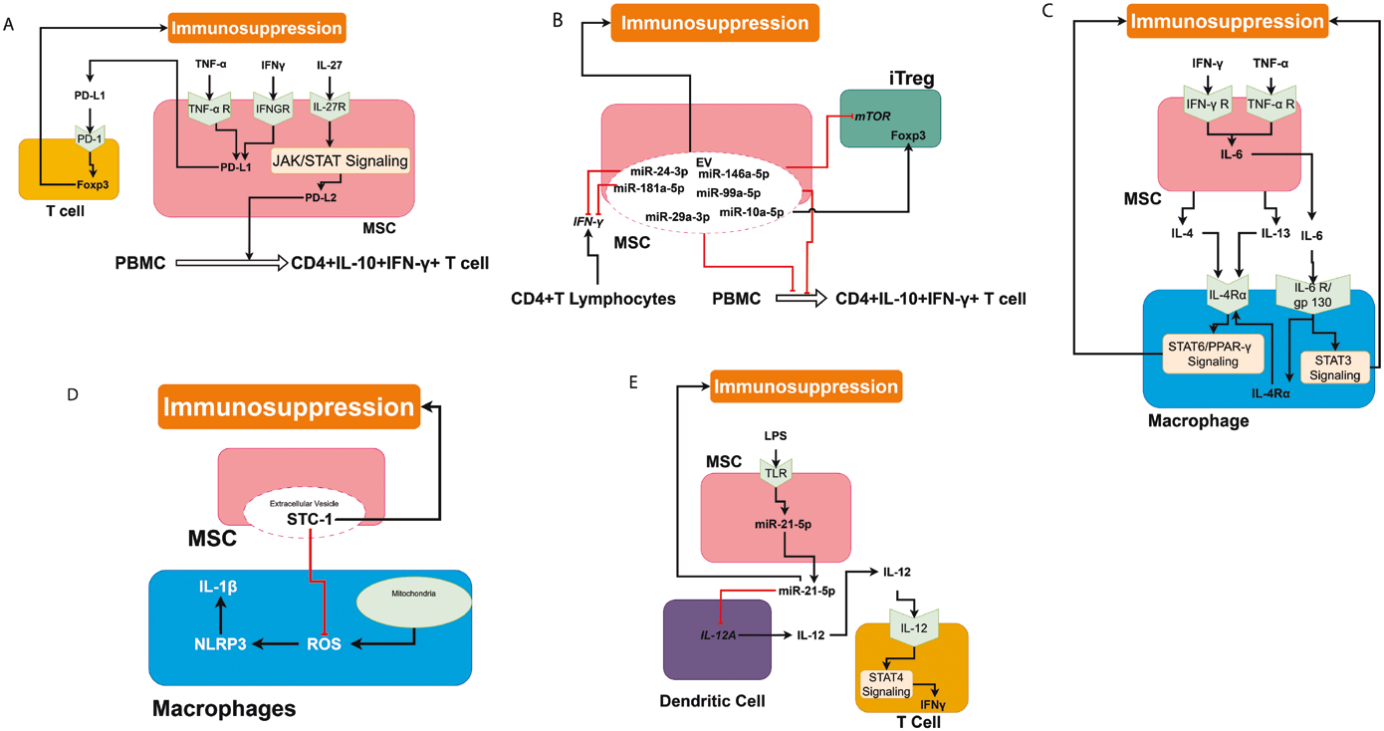
MSC interactions leading to immunosuppression. (A) TNF-α and IFN-γ enhance the expression of PD-L1, which binds to its receptor on T cells to induce Foxp3; (B) EVs in MSC contain microRNAs (miR), which promote immunosuppression mechanisms; (C) IFN-γ and TNFα induces IL-6 expression in MSCs. This IL-6 binds to its receptors on the macrophages and induces STAT3 signaling and induces immunosuppression. The binding of IL-6 to its receptor also expresses IL-4Rα. This increases the IL-4/ IL-4Rα and IL-13/ IL-4Rα signaling and induces immunosuppression; (D) Stanniocalcin-1 in the MSC-EV inhibits the ROS and thus inhibits the NLRP3 inflammasomes; and (E) The binding of LPS TLR4 on MSCs results in the production of miR-21-5p, which inhibits IL-12. IL-12 is an important inducer of IFNγ.

**Figure 5.**
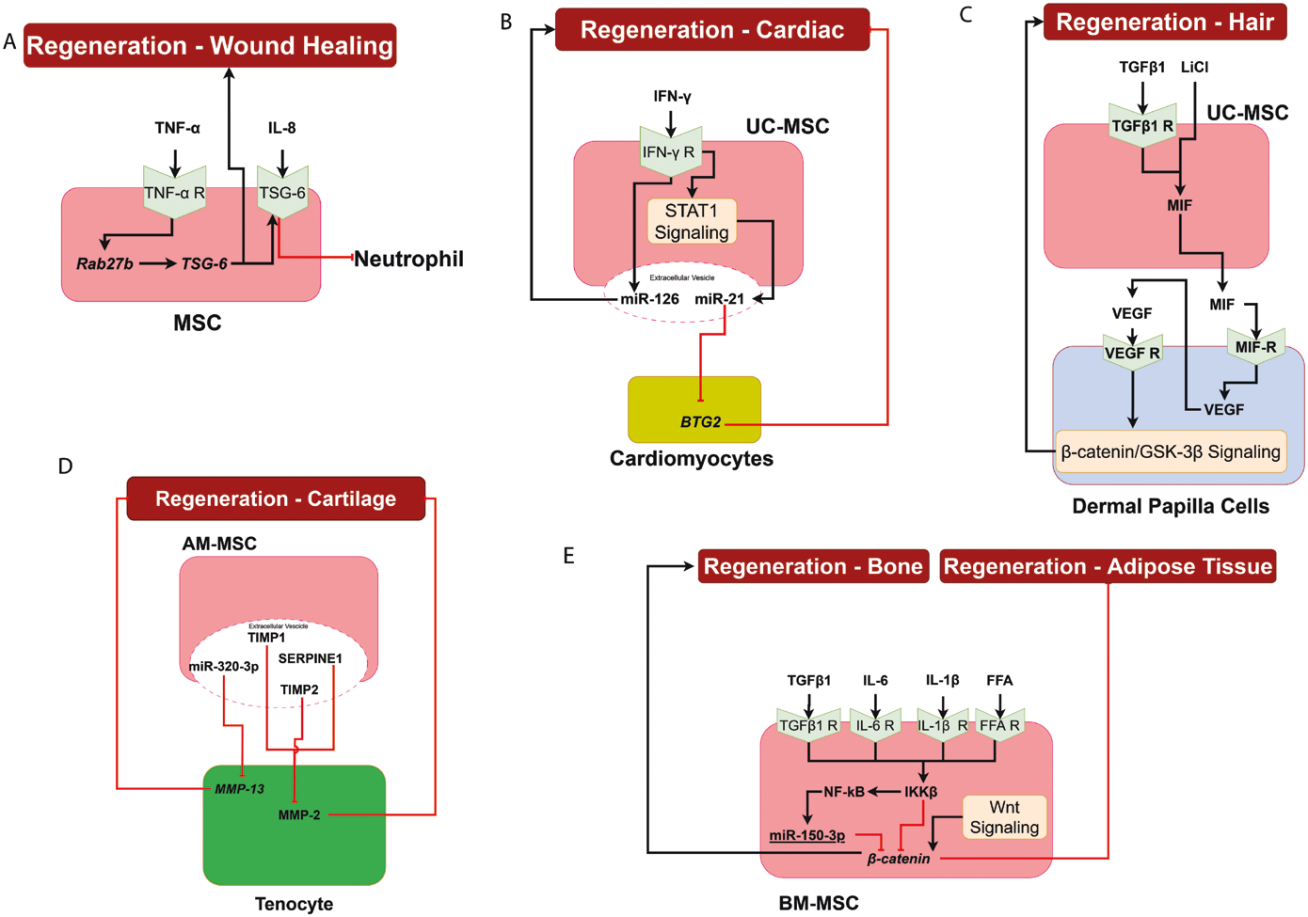
MSC interactions leading to tissue regeneration. (A) TNF-α induces TSG-6 in MSCs. IL-8 binds to TSG-6 to inhibit neutrophil extravasation and promotes wound healing; (B) IFN-γ induces the expression of miR-21 and miR-126 in MSCs. Upregulation of miR-126 promotes cardiac tissue regeneration. Upregulated miR-21 inhibits antiproliferation factor 2 (BTG2) expressions in the cardiomyocytes; (C) Treating MSCs with TGF-β1 and LiCl results in the secretion of MIF. MIF binds to its receptor on the DPCs and induces the expression of VEGF. VEGF through Wnt signaling induces hair regeneration; (D) Extracellular vesicles in AM-MSCs contain SERPINE1, TIMP, TIMP2, and miR-320-3p, all of which induce cartilage regeneration through inhibiting MMPs in tenocytes; and (E) Inflammatory cytokines induce the expression of IKK-β and NF-κb in MSCs, which induces miR-150-3p. IKK-β and miR-150-3p are inhibitors of β-catenin. β-catenin induces osteogenesis and inhibits adipogenesis.

**Figure 6.**
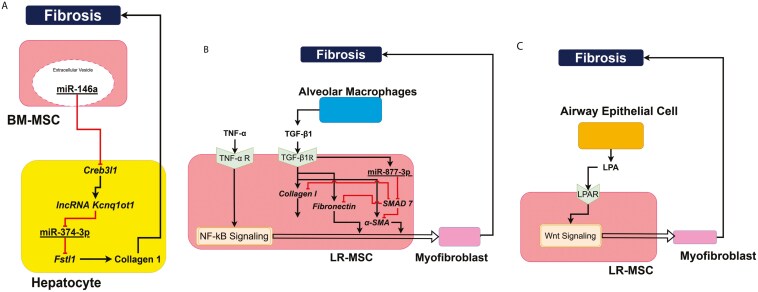
MSC interactions leading to fibrosis. (A) miR-374-3p in the BM-MSC EV inhibits lncRNA Kcnq1ot1 in hepatocytes, which promotes fibrosis in the liver; (B) TGF-β induces miR-374-3p in MSCs, which inhibits SMAD-7 which is an inhibitor of collagen I, fibronectin, and αSMA. TNF-α induced NF-kB is involved in the differentiation of MSCs into myofibroblasts along with other factors such as collagen I, fibronectin, and αSMA; and (C) LPA from injured lung cells induces the differentiation of lung resident MSCs to myofibroblasts by inducing Wnt signaling.

MSC microenvironmental cells—NK cells, DCs, macrophage, T cell, myofibroblasts, chondrocytes, epithelial cells, adipocytes, and cardiomyocytes—communicate with each other through a multitude of molecular pathways as shown in the interactome in Figure 3. The details of some of the key biochemical interactions from the Molecular Pathways layer in Figure 2 that affect immunosuppression, regeneration, fibrosis, and antifibrosis are shown in Figure 3. The legend for the symbols in Figures 3–6 is presented in the Supplementary Information (Table S1).

### Interactome of cellular biochemical pathways in molecular systems architecture subsystems

An additional eleven subsystems of the interactome have been identified, and the details are provided in Supplementary Information. These subsystems include: NLRP3 signaling (Figure S3); stanniocalcins signaling (Figure S4); CD40/CD40-L signaling (Figure S5); ICAM-1/CD-43 signaling (Figure S6); VEGF/VEGR3 signaling (Figure S7); thrombospondin-1 (TSP-1) signaling (Figure S8); IFN-γ/TNF-α signaling (Figure S9); miR-218 signaling (Figure S10); TGF-β signaling (Figure S11); WNT5A signaling (Figure S12); and, retinoic acid signaling (Figure S13).

### Crosstalk signaling across cellular components of the MSC microenvironment

This section is organized as follows: (1) immunomodulating function of MSCs, (2) regenerative capacity of MSCs, (3) fibrosis and MSCs, (4) novel mechanisms of MSCs, and (5) MSCs in clinical therapy.

#### Immunomodulating function of MSCs

The immunomodulatory capacity of MSCs is dependent on the presence of PGE2, enabling them to inhibit T cells and interact with macrophages.^23^ Additionally, MSCs secrete soluble factors such as TGF-β, NO, and IDO, which modulate the immune system by inhibiting T-helper 1 (Th1) and Th17 cell activation and proliferation, reducing inflammatory cytokines like IFN-γ and IL-17, suppressing DC activation, blocking monocyte differentiation, and reducing IL-12 production.^38^ Some of the key interactive signaling pathways between MSCs and immune cells are described below.

#### MSC–T cell interactions

Programmed death-1, a negative costimulatory molecule on activated T cells, can trigger T cell death by interacting to its ligand PD-L1/2 or upregulating suppressor gene expression.^56^ In MSCs, TNF-α and IFN-γ can increase the expression of immunoregulatory proteins such PD-L1,^20,57^ which may inhibit T cell activation via the PD-L1/PD-1 pathway.^58^ The transmembrane PD-L1, but not the soluble PD-L1, is reported to play a more critical role in immunosuppressive activity of MSCs.^59,60^ For MSC-mediated immunosuppression of T cells to occur, the PD-1/PD-L1 pathway is necessary as PD-L1 and PD-L2 released by MSCs could control Foxp3 expression. IL-27-induced expression of PD-L2 in human placenta MSCs (hPMSCs) through the Janus kinase/signal transducer and activator of transcription (JAK/STAT) pathway. When PD-L2 producing MSCs were cocultured with peripheral blood mononuclear cells, they induced production of CD4 + IL-10 + IFN-γ+ T cell subsets in activated PBMC.^40^ Schematics of interactions among T cells and MSCs are illustrated in Figure 4A.

miRs from the EVs of MSCs play a key role in immune cell function and polarization.^61^ While miR-29a-3p and miR-146a-5p induce development toward inflammatory Th1 cells, miR-24-3p and miR-181a-5p suppress IFN-γ production in CD4 cells. Targeting the mammalian target of rapamycin (mTOR), miR-99a-5p influences inducible Treg polarization, while miR-10a-5p causes Treg polarization by maintaining Foxp3 expression,^62^ resulting in an immunosuppressive environment. Schematics of miR regulation of T cell polarization are illustrated in Figure 4B.

#### MSCs–macrophage interactions

MSC-derived IDO, PEG2, miR-223, and IL-6 promote macrophage polarization toward the M2 phenotype, which speeds up wound healing.^63^ Stanniocalcin-2 (STC-2) has been demonstrated to modulate the action of MSCs by altering polarization of the macrophages from M1 to M2 phenotype.^44^ Mesenchymal stromal cells also promote the M1 to M2 phenotype transition of activated macrophages via soluble mediators such as IDO and PGE2.^64^ Mesenchymal stromal cells can produce insulin-like growth factor (IGF)-2 that endows maturing macrophages with anti-inflammatory properties.^42^

IFN-γ and TNF-α-primed MSCs greatly improve the phagocytic activity of M2 macrophages via IL-6^63^ through the IL-6/IL-4Rα/STAT6/PPAR-γ and IL-6/STAT3 signaling pathways. In the IL-6/IL-4Rα/STAT6/PPAR-γ pathway, IL-6 from MSCs upregulates the IL-4Rα receptor expression on the surface of macrophages, thereby upregulation and promotion of IL-4 and IL-13 binding to IL-4Rα receptors leading to activation of STAT6. The activated STAT6 directly binds to PPAR-γ, which in turn initiates transcription of genes needed for M2 polarization. In the IL-6/STAT3 pathway, which is inhibited in M1 macrophages but not M2 macrophages, IL-6 binds with IL-6R and glycoprotein 130 (gp130) and forms a polymer that activates STAT3; which in then enters the nucleus to induce genes that regulate macrophage polarization to the M2 phenotype.^63^ Interactions between MSCs and macrophages are illustrated in Figure 4C and D.

Mesenchymal stromal cells, specifically TMSCs can inhibit the activation of NLRP3 inflammasome in macrophages by the suppression of mitochondrial ROS generation and consequent IL-1β production via release of antioxidant STC-1 that is initiated by soluble factors from activated macrophages, thus leading to the attenuation of macrophage apoptosis.^30^

#### MSCs–T cell–DC interactions

DCs produce IL-12 that dominantly targets T cells by inducing T cell IFN-γ production through STAT4. When treated with MSC-EV, the highly expressed miR-21-5p in the MSC-EV IL-12A and inhibit IL-12p35 in DCs^65^ leading to decreased amounts of IL-12-p70, thus favoring the promotion of support Th2 phenotype over Th1 phenotype^65^ and leading to immunosuppression. These interactions are illustrated in Figure 4E.

### Regenerative capacities of MSCs

Mesenchymal stromal cells have been successfully used in various regenerative applications, including wound healing and regeneration of tissues in liver, heart, and cartilage, to name a few, due to their trans-differentiation capacity.^16,66^ Molecular mechanisms of MSC involvement in wound healing, cardiac tissue regeneration, hair regeneration, liver regeneration, cartilage regeneration, and bone regeneration are discussed in detail as follows. Some of the key interactive signaling pathways involved in the regenerative capacities of MSCs are described below.

#### Wound healing

Mesenchymal stromal cells regulate wound healing and cell growth through paracrine cytokine signaling.^67^ Pro-inflammatory cytokines such as TNF-α induce MSCs to secrete high levels of TSG-6, which mitigates the inflammatory responses of resident macrophages and inhibits neutrophil extravasation via its direct ligand, CXCL8 (IL-8), thereby preventing tissue damage during acute inflammation and promoting wound healing.^36^ The secretion of TSG-6, potentially facilitated by Rab27b, is crucial for MSC immunopotency.^68^ Schematics of the role of MSCs in wound healing are illustrated in Figure 5A.

#### Cardiac tissue regeneration

Exosomes secreted from IFN-γ-treated MSCs show protective effects against myocardial injury. Among the soluble factors in these exosomes, miR-21, miR-125b, and miR-126 play a critical role in cardiac tissue regeneration. miR-21 is significantly upregulated in IFN-γ-primed MSC exosomes through STAT1 signaling.^69^ miR-21 inhibits the expression of B-cell translocation gene antiproliferation factor 2 (BTG2), thus downregulating the cardiac cell apoptosis. Exosomes derived from hypoxic conditions exerted stronger inhibition of cell death through miR-125b. Hypoxia-treated MSC-derived exosomes exhibit more effective cardio-protection mediated by urothelial cancer-associated 1 and miR-125b. Apart from miR-21, miR-126 is also present in higher amounts in IFN-γ-treated MSC exosomes. miR-126 carried by exosomes promotes angiogenesis and attenuates apoptosis.^69^ Schematics of the role of MSCs in cardiac tissue regeneration are illustrated in Figure 5B.

#### Hair growth

When UC-MSCs are treated with a combination of TGF-β1 and lithium chloride (LiCl), they secrete macrophage migration inhibitory factor (MIF). The MSC-derived MIF regulated the secretion of VEGF in dermal papilla cells (DPCs). The secretome derived from UC-MSCs has been shown to improve hair growth in paracrine mechanism, whereas MIF improved hair growth through mechanisms such as VEGF-induced β-catenin p-glycogen synthase kinase (GSK)-3β signaling pathway.^45^ Schematics of the role of MSCs in hair growth are illustrated in Figure 5C.

#### Cartilage regeneration

High levels of tissue inhibitor of metalloproteinases (TIMP) 1 and 2 are present in the AM-MSC secretome. The expression of TIMP1 was shown to inhibit extracellular matrix (ECM) degradation by metalloproteinase 2 (MMP2). TIMPs regulate ECM remodeling by directly inhibiting MMPs. Other abundant secretome factors involved in ECM remodeling are serpin family E member 1 (SERPINE1), which is an inhibitor of proteases. SERPINE1 is upregulated in a transient manner post-tendon injury to offset the matrix remodeling and mediates fibrotic adhesions by suppressing MMP activity. Additionally, the AM-MSC secretome also contains ECM-related factors that play a regenerative and protective role in tendons and cartilage.^62^ Schematics of the role of MSCs in cartilage regeneration are illustrated in Figure 5D.

#### Bone regeneration

BM-MSCs can differentiate toward adipocytes or osteoblasts under different stimulations.^18^ Osteogenic differentiation of BM-MSCs is more complicated than their adipogenic differentiation. A number of adipogenic factors are involved in the regulation of gene expression during the osteogenic differentiation. Among these, peroxisome proliferator-activated receptor γ (PPARγ) plays a crucial role by promoting adipogenic differentiation and inhibiting osteogenic differentiation via controlling downstream adipogenic and osteogenic genes. Increased mRNA expression of Wnt signaling pathway-related regulators such as, β-catenin, frizzled class receptor (FZD)3, Runx2, and Wnt5A have been shown to upregulate the osteogenic differentiation of human BMSCs.^18^

IKKβ as a key molecular switch that regulates MSC fate. Activation of IKKβ leads to phosphorylation and ubiquitination of IκB, and the nuclear translocation of nuclear factor kappa B (NF-κB) for the subsequent gene expression regulation. Additionally, IKKβ signaling in MSCs through an NF-κB-independent mechanism has been shown to favor adipogenesis and suppress osteogenesis. Wnt/β-catenin signaling is essential for osteoblast formation from MSCs but inhibits adipocyte differentiation. Free fatty acid-elicited IKKβ activation enhanced adipogenesis and inhibited osteogenesis of MSCs. NF-κB can upregulate miR-150-3p that targets β-catenin to inhibit osteogenesis in MSCs.^18^ Schematics of the role of MSCs in osteogenesis are illustrated in Figure 5E.

### Dual role of MSCs in fibrosis

Mesenchymal stromal cells play a dual role in fibrosis. In the lung, LR-MSCs have been shown to cause fibrosis,^66^ whereas in the liver, BM-MSCs are antifibrotic.^66^ Some of the key mechanisms implicated in the role of MSCs in liver and lung fibrosis are described in detail below.

#### Liver fibrosis

In the liver, BM-MSCs show antifibrotic potential. BM-MSCs reduce liver fibrosis via immunosuppressive and anti-inflammatory activities such as inhibition of NK cells and DCs, Th1 cell proliferation, and activation of M2 macrophages and Th2 cells.^19,55^ Activated M2 macrophages inhibit fibrosis. BM-MSC transplantation in liver significantly downregulated the mRNA expression levels of the fibrosis-related genes such as α-SMA, TGF-β1, collagen-4, and collagen-1. On the other hand, human embryonic stem cells (HeSCs)-derived TGF-β1 accelerated liver fibrosis, whereas myofibroblasts differentiated from HeSCs expressed pro-fibrotic α-SMA. Pro-inflammatory M1 macrophages activated of HeSCs leading to fibrosis, whereas BM-MSCs suppressed the activation of HeSC and upregulated apoptosis of hematopoietic stem cells,^19^ thereby mitigating fibrosis. The knockdown of potassium voltage-gated channel subfamily Q member 1 opposite strand/antisense transcript 1 (Kcnq1ot1) alleviated liver cirrhosis. BM-MSCs alleviate liver cirrhosis by modulating the Creb3l1/long noncoding(lnc)RNA Kcnq1ot1/miR-374-3p/Follistatin-related protein 1 (Fstl1) signaling pathway.^55^ Recent findings suggested that miR-146a, a known suppressor of CREB3l1, is present in the BM-MSC secretome and may explain alleviation of liver cirrhosis by BM-MSCs.^70^ Schematics of the role of MSCs in liver fibrosis are illustrated in Figure 6A.

#### Pulmonary fibrosis

Mesenchymal stromal cells play a pro-fibrotic role in the lungs during lung tissue injury. Upon injury, lung resident MSCs get activated and are differentiated into myofibroblasts, leading to increased α-SMA expression and collagen secretion, thereby causing diseases such as pulmonary fibrosis and bronchiolitis obliterans (popcorn lung).^15^ Chronic inflammation upregulates the pro-inflammatory factors (eg, TNF-α and IL-6) and pro-fibrotic factors (eg, TGF-β) in the idiopathic pulmonary fibrosis initial stages.^15^ TNF-α-initiated NF-κB signaling induces the differentiation of LR-MSCs to myofibroblasts and leads to lung fibrosis. The differentiation of LR-MSC into myofibroblasts is induced by the pro-fibrotic cytokine TGF-β1. In pulmonary fibrosis, most of TGF-β1 is secreted by injured alveolar epithelial type II cells (ATII) and alveolar macrophages. miR-877-3p expression is upregulated in LR-MSCs once they are exposed to TGF-β1, leading to upregulation of myofibroblasts differentiation marker such as fibronectin, αSMA, and collagen I. miR-877-3p also inhibits the Suppressor of Mothers Against Decapentaplegic (SMAD)-7, which inhibits the expression of TGF-β-induced collagen I and αSMA, thus promoting fibrosis through LR-MSC differentiation to myofibroblasts.^15^ Schematics of the role of MSCs in lung fibrosis are illustrated in Figure 6B and C.

Lung injury stimulates the production of bioactive lipid LPA, which activates LPA receptor 1 (LPAR1) on LR-MSCs, leading to migration and differentiation of LR-MSCs into fibroblasts^15^ in a β-catenin-dependent pathway. LPA upregulates GSK-3β phosphorylation, which initiates nuclear translocation of β-catenin and expression of genes governing cellular migration and differentiation of LR-MSCs into myofibroblasts, which overexpress genes such as Wnt8b, Wnt10a, and deSUMOylation enzyme SUMO-specific peptidase 1 (SENP1).^15^

### Recent discoveries in novel mechanisms of MSCs

Key novel molecular pathway mechanisms identified in the MSCs in recent literature are described in detail below.

#### Mitochondrial transfer

Under certain conditions, such as hypoxia, injury to neighboring cells, and inflammation, MSCs have been shown to transfer their mitochondria to initiate tissue repair mechanisms.^71^ For example, MSCs have been observed to transfer mitochondria to the islet cells through tunneling nanotubes (TNTs) composed of actin^22^ leading to higher oxygen consumption and enhanced insulin secretion in islet cells, indicating that MSCs augmented islet cell function leading to increased ATP production during hypoxic conditions. The protective properties of MSCs towards islet cells during hypoxia were observed without any direct contact between these cells indicating the role for MSC-secreted factors or MSC-conditioned medium in improving the survival of islet cells. Exposure to hypoxia was accompanied by downregulation of B-cell leukemia/lymphoma 2 protein-associated X (Bax) mRNA expression.^22^

#### Migrasome

A newly discovered organelle in MSCs, migrasomes are implicated in intercellular communication,^17^ homeostatic maintenance, and tissue repair.^72^ Migrasomes are part of the extracellular vesicle system and are generated on the retraction fibers while the cells are migrating.^73^ There are 3 key molecules involved in the initiation and stabilization of migrasomes—integrins, tetraspanins, and cholesterol.^74^ Integrins connect the migrasomes to the extracellular membrane of MSCs at the migrasome formation site, whereas tetraspanins and cholesterol help stabilize the migrasome to the retraction fibers.^74,75^ The migrasomes contain chemokines such as CXCL12, which supports the intracellular communication role for the migrasomes in the hematopoietic stem cell niche.^17^ A recent animal study demonstrated that AD-MSCs generated migrasomes containing CXCL12 via a CXCL12/CXC chemokine receptor (CXCR) 4-dependent pathway, which further recruits more AD-MSCs to the site of injury that initiated adipose tissue regeneration.^72^

### Clinical applications of MSCs

Mesenchymal stromal cells are emerging as a potential therapeutic agent for various diseases, including tissue transplant,^76^ cardiovascular diseases,^77^ infectious diseases,^78^ reproductive diseases,^79^ liver diseases,^80^ pulmonary diseases,^81^ musculoskeletal diseases,^82^ and autoimmune diseases.^83^ Some of the key therapeutic applications are summarized below.

#### Viral infections

The inflammatory hyper-response can lead to cytokine storm, which in turn leads to damage to the pulmonary tissue damage, multiple organ failure, acute respiratory distress syndrome, dysfunctional air exchange, and in some cases death in viral infections such as coronavirus disease 2019 (COVID-19).^84^ UC-MSC infusions were reported to be well-tolerated when infused in COVID-19 patients.^81^ Moreover, MSC infusion was found to improve pulmonary function,^85^ reduce inflammatory biomarkers,^86^ and decrease mortality and recovery time.^87^ Some MSC-related adverse events were recorded, including shivering, liver dysfunction, fever, headache, facial flushing, and heart failure.^44^

#### Bacterial infections

Dental pulp multipotent MSCs have shown direct antibacterial activity against several bacteria, including *Streptococcus mutans*, *Staphylococcus aureus*, *Fusobacterium nucleatum*, *Escherichia coli*, and *Lactobacillus acidophilus*.^88^ The secretory factors from the dental pulp MSCs contain cytokines such as IL-6 and IL-8 and growth factors such as angiopoietin-1 and hepatocyte growth factor (HGF), which promote antibacterial activity of MSCs owing to the bactericidal regions in their amino acid sequence.^88,89^

#### Androgenic alopecia

Androgenic alopecia is one of the most common types of hair loss, which is caused by high concentrations of dihydrotestosterone (DHT) in the scalp, leading to progressive loss of the hair follicles.^90^ Extracellular vesicles from AD-MSCs carry miR-122-5p, which was shown to promote hair follicle growth via downregulating DHT inhibition of hair follicles, upregulating the expression of β-catenin, and downregulating the expression of TGF-β1.^90^ In DPCs, DHT downregulated the antiapoptotic protein BCL2 expression, whereas it upregulated pro-apoptotic protein Bax. AD-MSC-derived miR-122-5p has been shown to exert pro-angiogenic properties via activation of VEGF.^91^

#### Fertility treatment

Endometrial-derived MSCs (EnMSCs), BM-MSCs, and UC-MSCs have been shown to have substantial therapeutic efficacy in treatment of infertility.^92^ BM-MSCs secrete antifibrosis and antiapoptosis cytokines such as IGF, VEGF, and HGF that help ovarian restoration. BM-MSC transplantation effectively repaired endometrium by upregulating the expression of the estrogen and progesterone receptors.^93^ Similarly, AD-MSCs have been shown to improve ovarian dysfunction by increasing the rate of maturing follicles, oocyte number, and corpora lutea by secretion of specific paracrine cytokines and changing gene expression.^94^

AD-MSC treatment also decreased apoptosis in granulosa cells. AD-MSCs can differentiate into endometrial cells, and their transplantation has been shown to repair the endometrial injury.^95^ Human UC-MSC-derived HGF can potentially trigger the activation of primordial follicles. The intra-testicular administration of MSCs or their secretome also facilitated the restoration of spermatogenesis through the paracrine factors released by MSCs. In vitro studies showed that the VEGF in the MSC secretome prompted Leydig cells to produce testosterone.^96^

## Discussion

This study hypothesized that the development of a systems biology approach—a molecular systems architecture of the MSC interactions with its microenvironment—may provide a systems-level understanding of MSCs’ potential as a therapeutic option for complex diseases. The results from this effort provide: a multilayered visual map of biomolecular interactions between MSCs and their microenvironment, insights into the molecular intra- and intercellular crosstalk in the MSC microenvironment, identification of molecular species that can be used as a therapeutic target for MSCs, and a foundation to create *in silico* computational models to provide quantifiable predictions that may lead to the development of combination therapeutics, clinical strategies to improve therapeutic efficacy, and the avoidance of adverse effects.

The first result from this study is a molecular systems architecture that is multilayered and provides a visual map of interactions among the microenvironmental cells that include the MSCs, tenocytes, cardiomyocytes, alveolar epithelial cells, hepatocytes, and immune cells, including monocytes, macrophages, T cells, Treg cells, and DCs, as shown in Figure 3. This map enables visualizing the effect of any perturbations in molecular interactions in one cell may impact the biological functions in a neighboring cell, leading to either inhibition or upregulation of a biological process such as immunosuppression, regeneration, and fibrosis that are modulated by MSCs. The molecular systems architecture is currently being converted to a publicly accessible web-enabled visual resource, to be hosted at this URL: https://integrativesystems.org/open-science-institute/stem-cell-research/web-enabled-systems-architecture/, that enables the user not only to interact directly with the architecture but also provide feedback and critiques, which will be reviewed, updated when necessary, and curated.

Beyond its visual aspect, the molecular systems architecture also provides a systems view of cellular crosstalk across the 10 different cells of MSC microenvironment. The molecular systems architecture identified the following 5 crosstalk signaling pathways between MSCs and the immune cells that establish the immunosuppressive role of MSCs: PD-1/PD-L1 signaling across MSCs, monocytes, and T cells; MSC extracellular vesicle-derived microRNA regulation of T cells and Tregs; IL-13/IL-4 signaling pathway between MSCs and macrophages; STC-1 signaling pathway between MSCs and macrophages; and, IL-12 signaling pathways across MSCs, DCs, and T cells. Across MSCs, neutrophils, cardiomyocytes, DPCs, tenocytes, and bone cells, the architecture revealed 5 crosstalk signaling pathways implicated in the regenerative role of MSCs: TSG-6 signaling pathway across MSCs and neutrophils; IFN-γ signaling pathway between MSCs and cardiomyocytes; TGF-β signaling pathway between UC-MSCs and DPCs; MMP2/13 signaling pathway between AM-MSCs and tenocytes; and TGF-β/IL-6/IL-β/FFA signaling pathways within BM-MSCs. Three crosstalk signaling pathways were identified from interactions between MSCs, hepatocytes, alveolar macrophages, and airway epithelial cells implicated in pro- and antifibrotic role of MSCs: the Fstl1 signaling pathway between BM-MSCs and hepatocytes; TGF-β signaling across LR-MSCs and alveolar macrophages; and, the LPA signaling pathway between LR-MSCs and airway epithelial cells. These insights provide an integrative, whole systems understanding of the MSC microenvironmental interactions, elucidating the role of MSCs in immunomodulation, regeneration, and pro-/antifibrosis.

The molecular systems architecture developed herein has also provided potential therapeutic targets for applications of MSC-derived secretory molecules. Across the cells of MSC microenvironment, targets for the MSCs-derived secretory molecules were identified that can either inhibit or activate the biological processes of immunosuppression, regeneration, and pro-/antifibrosis using the systems architecture, itemized in Table 1, and categorized according to the biological process that they affect. A total of thirteen molecular targets were identified across the MSC microenvironmental cells. Among these thirteen, one was identified in hair follicle cells, 3 in oocytes, one in DPCs, and one in tenocytes that affect regeneration; one target was identified in DCs, two in T cells, one in neutrophils, and one in macrophages that affect immunosuppression; and, finally, one target was identified in hepatocytes that affects fibrosis.

**Table 1.** Summary of potential therapeutic molecular targets.

MSC-derived molecule	Molecular target	Cell type	Biological process	Reference
miR-122-5p	Dihydrotestosterone (DHT)	Hair follicle cells	Regeneration	^91^
miR-21-5p	IL-12A	Dendritic cells	Immunosuppression	^65^
IGF, VEGF, HGF	Estrogen and progesterone receptors	Oocyte	Regeneration	^97^
HGF	c-Met	Oocyte	Regeneration	^97^
TSG-6	CXCL8	Neutrophils	Immunosuppression	^36,68^
STC-1	ROS	Macrophages	Immunosuppression	^30^.
STC-1	HO-1	T cells	Immunosuppression	^98^
ICAM-1	CD-43	T cells	Immunosuppression	^99^
MIF	VEGF	Dermal papilla cells	Regeneration	^45^
TIMP1 and TIMP2	MMP2	Tenocytes	Regeneration	^62^
miR-374-3p	lncRNA Kcnq1ot1 and Fstl1	Hepatocytes	Anti-Fibrosis	^55^

Abbreviations: CD-43, cluster of differentiation 43; c-Met, mesenchymal-epithelial transition factor; CXCL8, C-X-C motif chemokine ligand 8; Fstl1, follistatin like 1; HGF, hepatocyte growth factor; HO-1, heme oxygenase 1; ICAM-1, intercellular adhesion molecule 1; IGF, insulin growth factor; IL-12A, interleukin 12A; lncRNA Kcnq1ot1, long noncoding RNA KCNQ1 overlapping transcript 1; MIF, macrophage migration inhibitory factor; miR, microRNA; MMP2, matrix metalloproteinase 2; ROS, reactive oxygen species; STC-1, stanniocalcin-1; TIMP1, tissue inhibitor of metalloproteinases 1; TIMP2, tissue inhibitor of metalloproteinases 2; TSG-6, tumor necrosis factor-stimulated gene 6; VEGF, vascular endothelial growth factor.

The molecular systems architecture of the MSC microenvironment may enable the development of novel therapeutic options based on a systems approach. As an example, the molecular systems architecture can be utilized to determine how a combination of MSC-based therapeutics that either stimulate or inhibit different targets can impact various biological processes. Moreover, the visual map may demonstrate the positive or negative effects of targeting one or more molecular pathway in the MSC microenvironment on a particular biological process. Another example of an insight from the architecture is to understand how a therapeutic that works well for one target in a specific cell may have off-target effects in another microenvironmental cell, leading to an adverse side effect.

*In silico* mechanistic modeling of biological processes and diseases is being recognized as a useful preclinical drug discovery tool.^10,11,13,14,50^ The molecular systems architecture developed herein may serve as a foundation for creating predictive *in silico* models to represent the interactions in MSC microenvironment and can be utilized to answer research questions such as which of the targets from Table 1 can be more effective for modulation of MSC applications such as immunosuppression, regeneration, or pro-/antifibrosis? Moreover, these in silico models may prove useful to understanding efficacy and safety of single and multicombination treatments using MSCs, and finding the optimal dosages for these treatments. Employing a similar approach used in this study, a molecular systems architecture for pancreatic cancer was built previously and used to develop *in silico* models that successfully predicted a 2-drug combination, which was subsequently allowed by the US Food and Drug Administration.^14^ In another effort, systems architecture of osteoarthritis was utilized to build *in silico* models from which an optimal combination of 2 flavonoids that targeted the pain and inflammation molecular pathways was developed.^13^ Therefore, the molecular systems architectures, such as the one developed in this study, provide a framework for developing tangible therapeutic solutions.

## Supplementary Material

sxaf042_suppl_Supplementary_Tables_S1-S3_Figures_S1-S13

## Data Availability

The authors confirm that the data supporting the findings of this study are available within the article and its Supplementary Materials.
