# Supplementary material for: A molecular systems architecture of the mesenchymal stromal cell microenvironment
Source: Stem Cells. 2025 Aug 22;43(9):sxaf042. doi: 10.1093/stmcls/sxaf042 (PMC12371236; doi:10.1093/stmcls/sxaf042)
Supplement: sxaf042_suppl_Supplementary_Tables_S1-S3_Figures_S1-S13 [file sxaf042_suppl_supplementary_tables_s1-s3_figures_s1-s13.docx]

**Supplementary Information**

**A Molecular Systems Architecture of the Mesenchymal Stromal Cell Microenvironment**

V.A. Shiva Ayyadurai^1, 2*^, Vishvatha Radhakrishnan^1, 2^, Prabhakar Deonikar^1, 2^_,_ Armand Keating ^3^

^1^Systems Biology Group, CytoSolve Research Division, CytoSolve, Inc., Cambridge, MA, USA

^2^Open Science Institute, International Center for Integrative Systems, Cambridge, MA, USA

^3^Institute of Medical Sciences, University of Toronto, Toronto, ON, Canada

**CytoSolve® Operating Guide Protocol Summary**

**Introduction to CytoSolve® System**

CytoSolve is a well-established computational systems biology framework of technology and processes that provides the capability to derive molecular mechanisms of action; to create quantitative and predictive models of those mechanisms; and, to employ the resultant models to simulate complex biomolecular phenomena (1–5). In neurovascular studies, the CytoSolve framework elicited and derived a multi-layered engineering molecular systems architecture integrating the anatomy of the neurovascular unit, molecular mechanisms, and disease to demonstrate the commonality of multiple neurovascular diseases as communication dysfunctions in common molecular signaling sub-systems and compounds (5).

In oncology, CytoSolve’s capability has been employed for the *in silico* modeling of pancreatic cancer to identify and optimize a multi-combination therapeutic that was subsequently allowed for clinical trials by the United States Food and Drug Administration (6) has been used to identify the molecular systems architecture of interactome in acute myeloid leukemia (AML) microenvironment (7) and has been independently recognized by leading cancer researchers as a platform for developing multi-combination therapies (8). In cardiovascular research, CytoSolve has been used to accurately model the release of nitric oxide (NO) production in endothelial cells subjected to shear stress (3).

In the area of plant biology, CytoSolve enabled the quantitative molecular systems understanding of C1 metabolism - a critical system of molecular pathways inherent to all plants, fungi and bacteria - to understand the systemic effects oxidative stress and genetic modification on C1 metabolism in soy (9–12). Recently, CytoSolve was used to discover and model the mechanisms of immunomodulatory effect of bioactive compound in green tea on organ transplant tolerance, (13), and elucidate effect of bioactive compounds from fruit, berry, vegetable (FBV) juice power on low grade chronic inflammation (14).

**CytoSolve® System Capabilities**

The method used in this study provides a scalable computational framework for modeling large-scale biological systems by dynamic integration of an ensemble of multiple molecular pathway models (2). This method enables the development of large-scale models of complex biological systems that span multiple temporal and spatial scales as well as across diverse domains. Rather than attempting to monolithically model systems of biochemical reactions, a distributed engineering systems approach – a relatively novel concept in systems biology– is employed that breaks a large scale biological system into an ensemble of smaller molecular pathway models that are computationally coupled. This approach makes the modeling of large-scale biological systems both tractable and scalable.

**Key Elements of CytoSolve® System Protocol**

There are six (6) steps that comprise the protocol to use the CytoSolve® system. Supplementary Figure S1 illustrates those steps of the protocol.


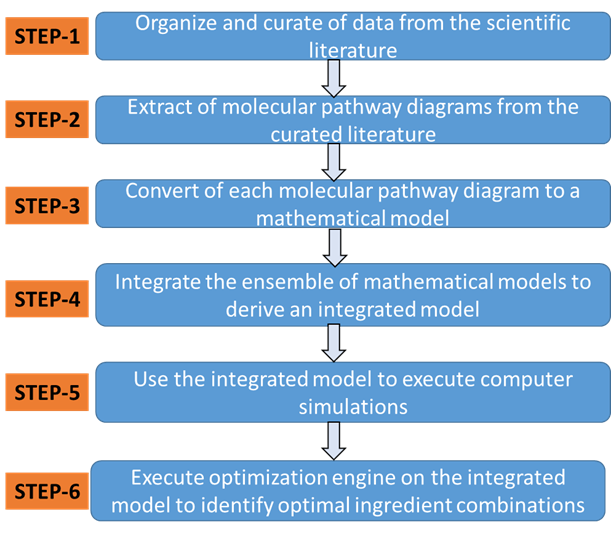


**Supplementary Figure S1.** CytoSolve® Protocol Overview. The six steps involved in the CytoSolve® protocol. Steps 1 and 2 relate to performing systematic literature review to identify molecular pathways and the biochemical parameters required for computational modeling. Steps 3 to 5 relate to construction of individual models, integration of individual models, and executing simulations using the integrated modes. Step 6 provides the option to employ CytoSolve® optimization engine to discover the optimal combination of inputs (ingredients/compounds) for a specific range of values of outputs (biomarkers associated with particular biomolecular functions).

The six (6) steps are listed below:

1) Organize and curate data from the scientific literature (§A4)

2) Extract molecular pathway diagrams from the curated literature (§A5)

3) Convert each molecular pathway diagram to a mathematical model (§A6)

4) Integrate individual mathematical models to derive an integrated model (§A7)

5) Use the integrated model to execute computer simulations to analyze the effect of ingredients on interest, individually as well as in combination (§A8)

6) Execute optimization engine on the integrated model to identify optimal ingredient combination (§A9)

**CytoSolve® Protocol for Organization and Curation of Data from the Scientific Literature**

This protocol step is the first step of the overall CytoSolve® Protocol indicated in Fig.A1. In this step, the scientific literature is searched to identify journal papers that contain research on the area of interest. For a particular area of interest, and *ingredients of interest*, molecular pathways for the area of interest, and the effect of ingredients of interest on those molecular pathways are identified and organized.

Four (4) specific steps are executed per the CytoSolve® Protocol to organize and curate the journal papers, as itemized below:

1. Create a list of Medical Subject Headings (MeSH) *keywords* to optimize recall and precision of peer-reviewed articles. The keywords are typically a list of words in combination with Boolean operators i.e. “AND”, “OR”, etc. The MeSH keywords are shown in Table S2
2. Search and retrieve the relevant peer-reviewed articles published during a specific *time period* from PubMed, Medline, and Google Scholar. These set of articles are stored as an “Initial Set” repository. The time period is a date range i.e. “January 2004 to January 2024,” etc.
3. Screen the titles and abstracts of articles in the Initial Set repository to identify most relevant articles based on our inclusion criteria. These set of articles are stored as the “Final Set” repository
4. Perform full-length review of peer-reviewed articles from the Final Set repository

Supplementary Figure S2 below represents the CytoSolve systematic bioinformatics literature review process inclusion criteria and categorization for MSC interactions with the microenvironmental cells.


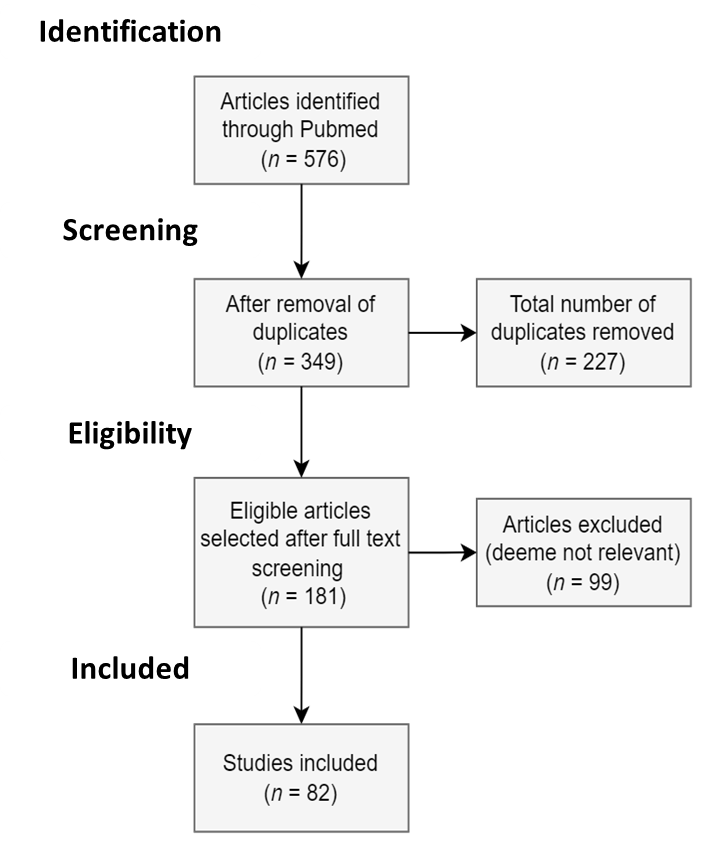


**Supplementary Figure S2.** PRISMA flow diagram. A total of 576 articles are identified; 227 duplicates were removed; 181 articles were eligible for review from which 99 were removed as they were deemed not relevant based on the inclusion criteria; and, 82 articles were included in the analysis.

**CytoSolve® Protocol for Extraction of Data from the Scientific Literature**

Journal articles in Group 1 are reviewed to gather data relevant to molecular pathways involved in the area of interest. The steps to extract and represent molecular pathways diagrammatically are itemized below:

1. Identify and extract:

a. chemical species involved in the molecular pathways

b. types of cells

c. cellular components (e.g. cytosol, mitochondria, nucleus, etc.) where the chemical species are present in each cell type

2. Identify and diagrammatically represent biochemical interactions

3. Interconnect biochemical reactions to create molecular pathway diagram in each cell type

Journal articles in Group 2 are reviewed to gather data relevant to pharmacokinetics of ingredients of interest.

Journal articles in Group 3 are reviewed to extract following information:

1. Reaction rate constants of biochemical reactions involved in molecular pathways
2. Molecular targets of ingredients of interest in the molecular pathways of interest

The kinetic parameters used in this study are derived using principles of Michaelis-Menten kinetics that are based on the steady-state approximation of the biochemical reactions (15,16).

**CytoSolve® Protocol for Setup of Individual In Silico Mathematical Models**

The steps to convert molecular pathway diagrams to mathematical models are itemized below:

1. Convert biochemical reactions involved in each of the molecular pathway into ordinary differential equations (mathematical expressions that describe the rate of change)

2. Represent each molecular pathway as a system of ordinary differential equations

3. Encode the system of differential equations in a computer software source code format known as Systems Biology Markup Language (SBML) (17) to construct a mathematical model for a particular molecular pathway.

4. Store each model as a separate SBML file

**CytoSolve® Protocol for Integration of Individual In Silico Mathematical Models**

In order to create an integrative quantitative model of the area of interest, it is necessary to mathematically couple the solutions across the ensemble of individual molecular pathway models. Such mathematical coupling is performed using the CytoSolve (4,13,18) computational engine, which is described in detail in Ayyadurai and Dewey, 2011 and Ayyadurai et al., 2022 (2,14). The computational architecture of CytoSolve enables the integration of plurality of molecular pathway models (2,19).

The steps to integrate the ensemble of individual mathematical models are listed below:

1. Upload individual SBML files, constructed in §A6 of Appendix A, to CytoSolve engine

2. Update the initial conditions for the molecular species in all the mathematical models in the graphical user interface

3. Update simulation period was specified in the graphical user interface

4. Review and confirm molecular species and reaction duplicates across all the individual models in the graphical user interface

5. Commence integration of individual models

For a computational system biology analysis wherein the simulation of biochemical reactions is being executed and the governing equations are well known, as is in this case, the error bounds are set prior to executing simulation (20,21). The error bounds are set to 10^-6^ prior to the execution of simulations. This means that the solutions to the governing equations used in the simulation of specific biochemical reactions must be within these error bounds. Therefore, *in silico* – computational – results from such simulations will not have error bars, which customarily appear in results reported from *in vitro* and *in vivo* experimental studies (22).

**CytoSolve® Protocol for Simulation of Integrated In Silico Mathematical Model**

The following steps are performed to execute the computer simulations:

1. Input biochemical reactions for interaction between ingredients of interest and molecular pathways involved in area of interest

2. Input the kinetic rate constants for each of the biochemical reaction

3. Input the initial concentrations for each of the molecular species in the biochemical reactions

4. Input the time period for the simulation of integrative models, and dose levels of ingredients of interest

5. Execute the integrative model under control conditions

6. Execute the integrative model in presence of ingredients of interest individually

7. Execute the integrative model in presence of the combination of ingredients

The steps for analyzing simulation output data are itemized below:

1. Export the raw data to Microsoft Excel

2. Extract the steady state levels of biomarkers

3. Plot steady state levels of biomarkers as a function of simulation time in presence and absence of ingredients of interest, individually as well as in combination.

**CytoSolve® Protocol to Execute Optimization Engine on the Integrated Model to Identify Optimal Ingredient Combination**

The following steps are performed to execute the optimization engine:

1. Simulate the integrative model in presence of ingredients of interest

2. Select “Optimize Results” on the CytoSolve® graphical user interface to initiate the optimization engine

3. In the optimization engine, choose “Objective” for the ingredients of interest and the biomarkers affected by the ingredients of interest. The objective is set to “optimize” for the ingredients of interest; “maximize” for the biomarkers that are related to positive outcome; and, “minimize” for the biomarkers that are related to negative outcomes.

4. Execute the optimization engine and record the results

The steps for analyzing simulation output data are itemized below:

1. Export the raw data to Microsoft Excel

2. Extract the values of ingredients of interest that have either minimized or maximized the biomarker

**CytoSolve® System Constraints**

Although the CytoSolve® Systems Biology framework provides a detailed mechanistic understanding that matches well with published clinical data, some of the model components’ parameters maybe derived from experiments using different cell types, as well as different experimental conditions such as variations in culture conditions that adds to the uncertainty of the model predictions (23). Such issues of parameter estimation, however, are not unique to this study. They are common to a number of cellular mathematical models (24) and do warrant further experimental investigation and validation.

In this section of the Supplementary Information, schematics for molecular pathway subsystems involved in MSC microenvironment in. There are eleven (11) molecular pathway subsystems. The schematics of these molecular pathway subsystems are shown below. Supplementary Table S1 provides the legend of the symbols used in the interactome figures. Molecular species including enzymes, proteins, ligands, and small molecules are represented in regular font, and mRNAs are represented in italic font.

**
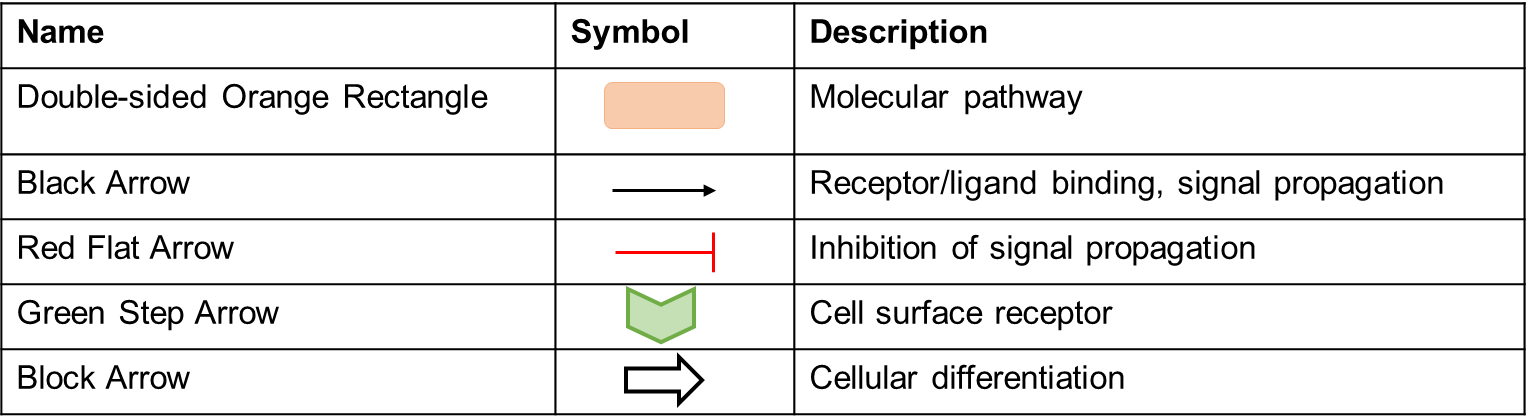
Supplementary Table S1.** Legend of symbols used in interactome figures.

**NLRP3 Signaling**


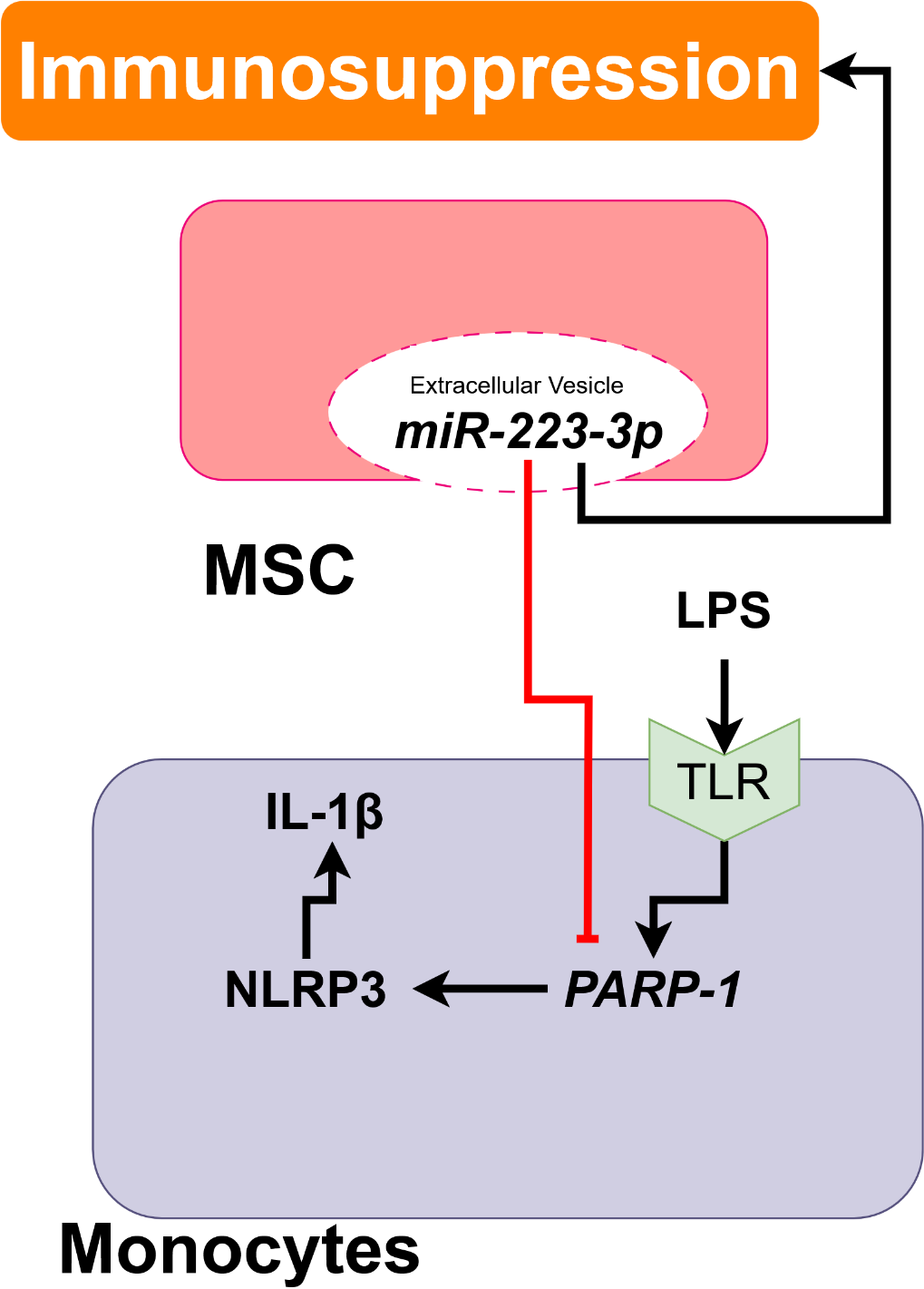


**Supplementary Figure S3: NLRP3 signaling in airway epithelium.** miR-223-3p from MSC-EV inhibits LPS induced PARP-1, which is important for the NLRP3 induced IL-1β, leading to immunosuppression.

**Stanniocalcin Signaling**


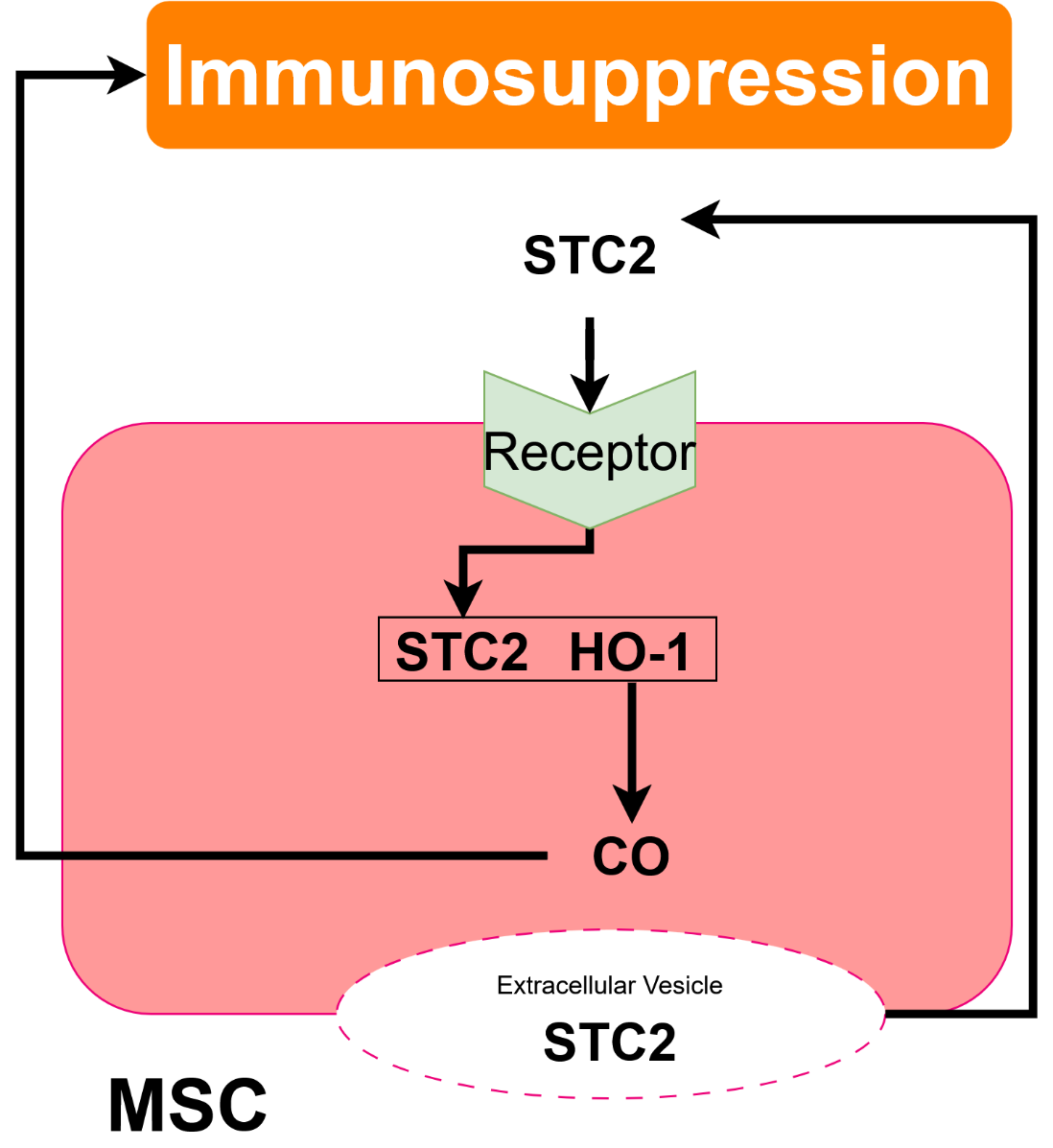


**Supplementary Figure S4: Stanniocalcins Signaling.** Stanniocalcin 2 co-localizes with heme oxygenase 1 (HO-1) in MSCs. HO-1 catalyzes the degradation of heme and releases CO which is key to the MSC-mediated suppression of allo-activated T cells.

**CD40/CD40-L Signaling**


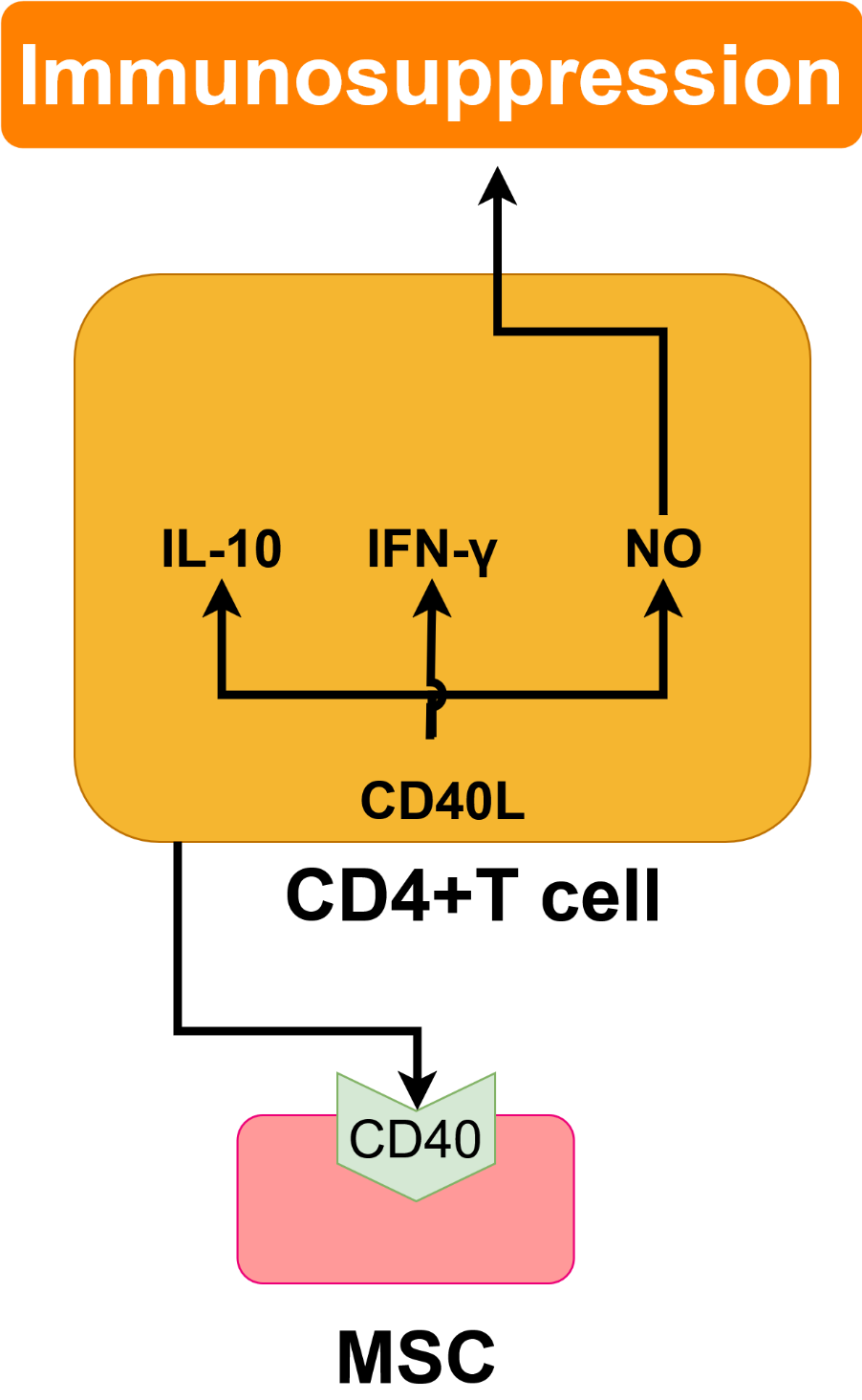


**Supplementary Figure S5: CD40/CD40-L Signaling.** CD40/CD40L Signaling between MSCs and T cells promote immunosuppression.

**ICAM-1/CD43 Signaling**


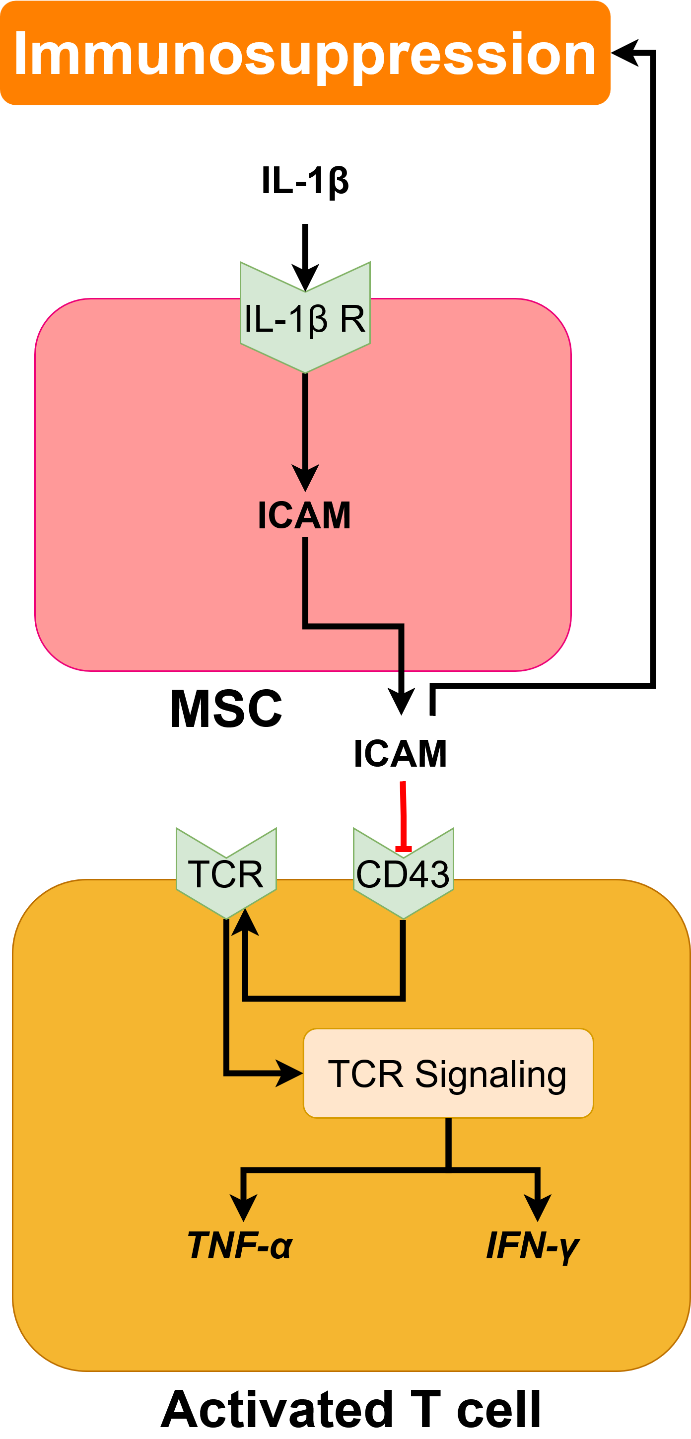


**Supplementary Figure S6: ICAM-1/CD43 Signaling.** IL-1β induced ICAM binds to the CD43 receptor and inhibits TCR signaling, leading to immunosuppression.

**VEGF/VEGFR3 Signaling**


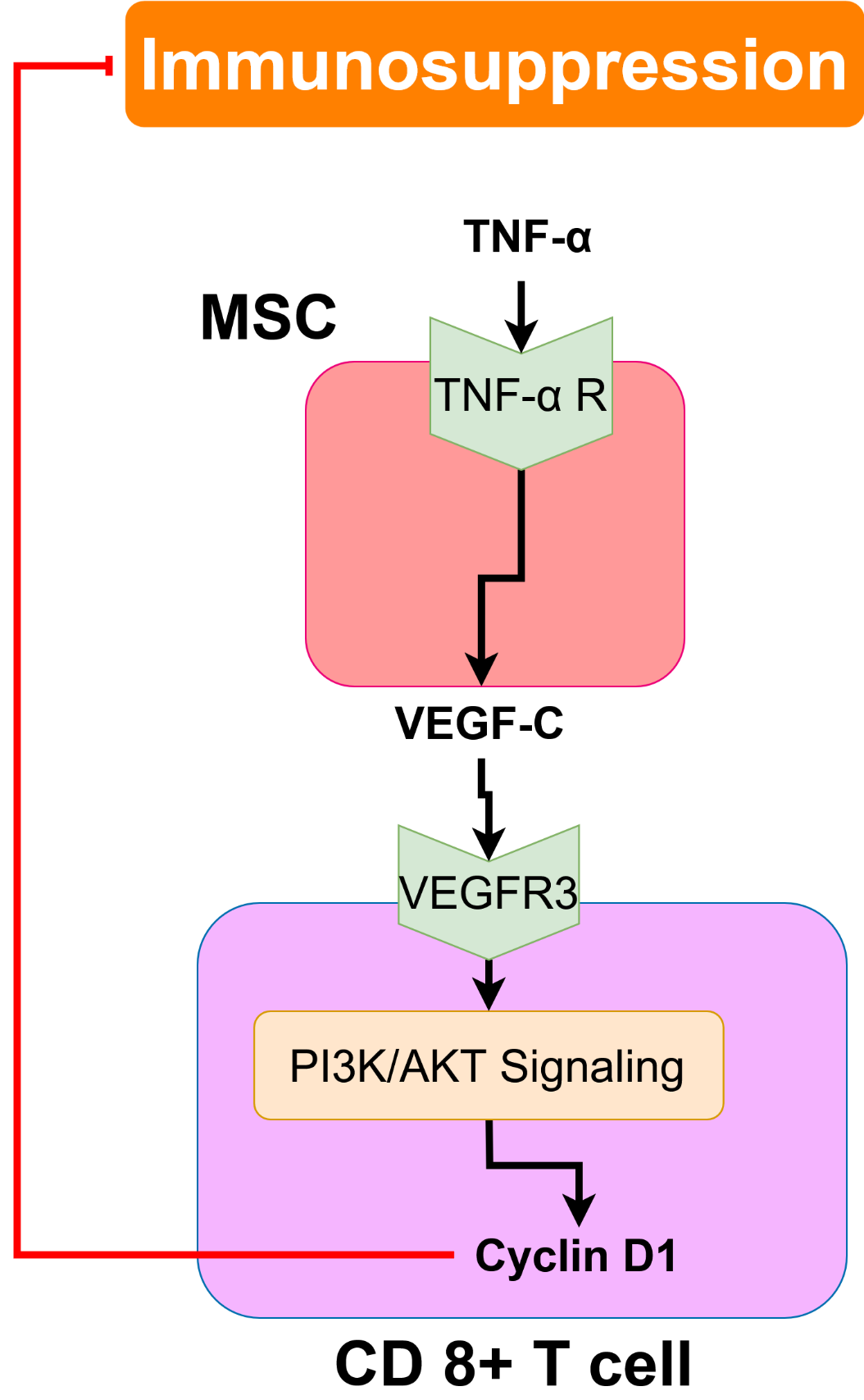


**Supplementary Figure S7: VEGF/VEGFR3 Signaling.** TNF-α induced VEGF-C binds to its receptor ion CD8+ T cells and induces cyclin D1 through pi3k/Stat signaling. This induces the proliferation of the T cells increases inflammation, thereby promoting immune response**.**

**Thrombospondin 1 (TSP-1) Induced Signaling**


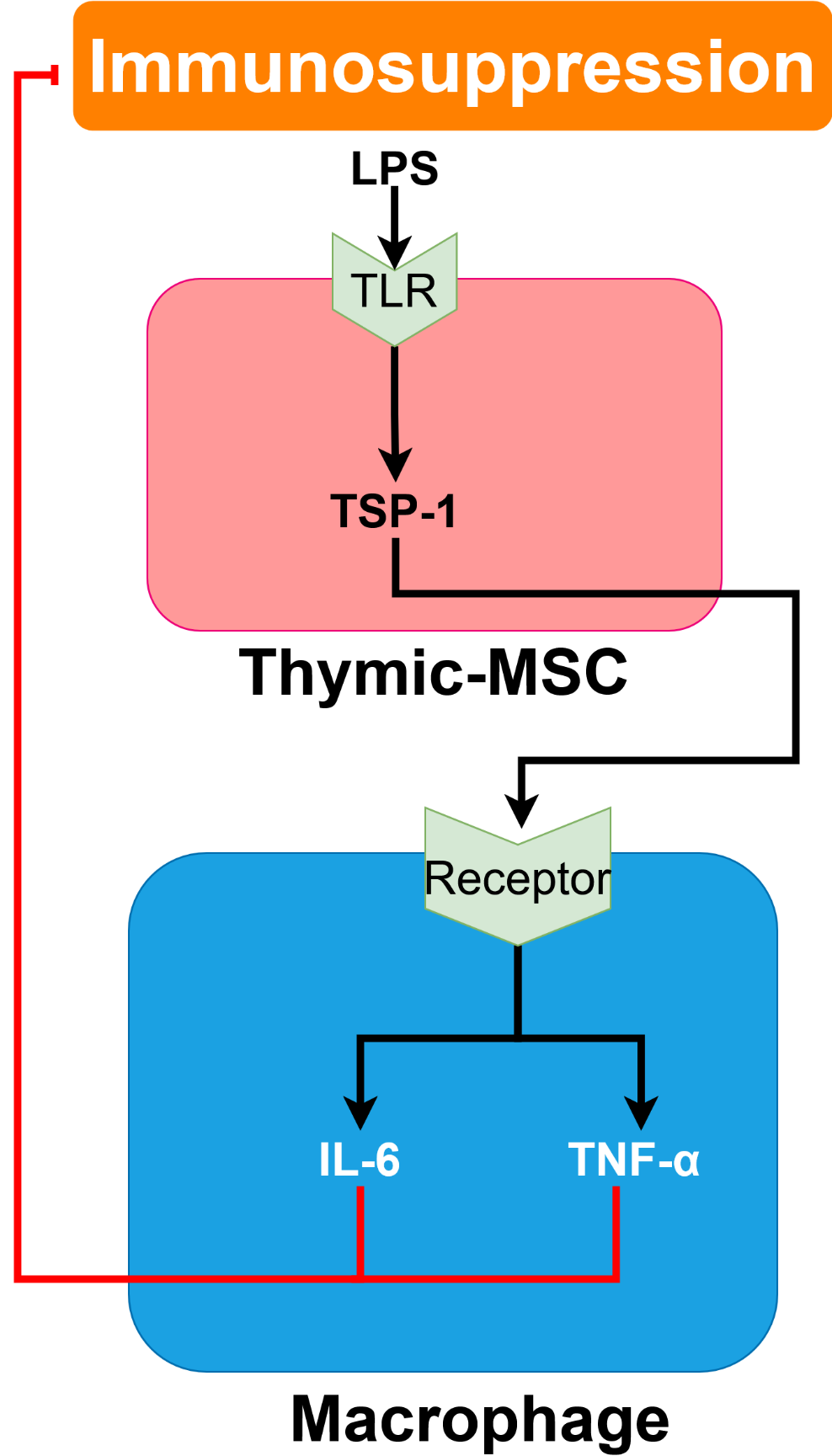


**Supplementary Figure S8: Thrombospondin (TSP-1) Induced Signaling.** Tonsil MSCs produce TSP-1, which binds to its receptors on macrophages. This results in the production of TNF-α and IL-6, thereby upregulating the immune response.

**IFN-γ/TNF-α Signaling in Wound Healing**


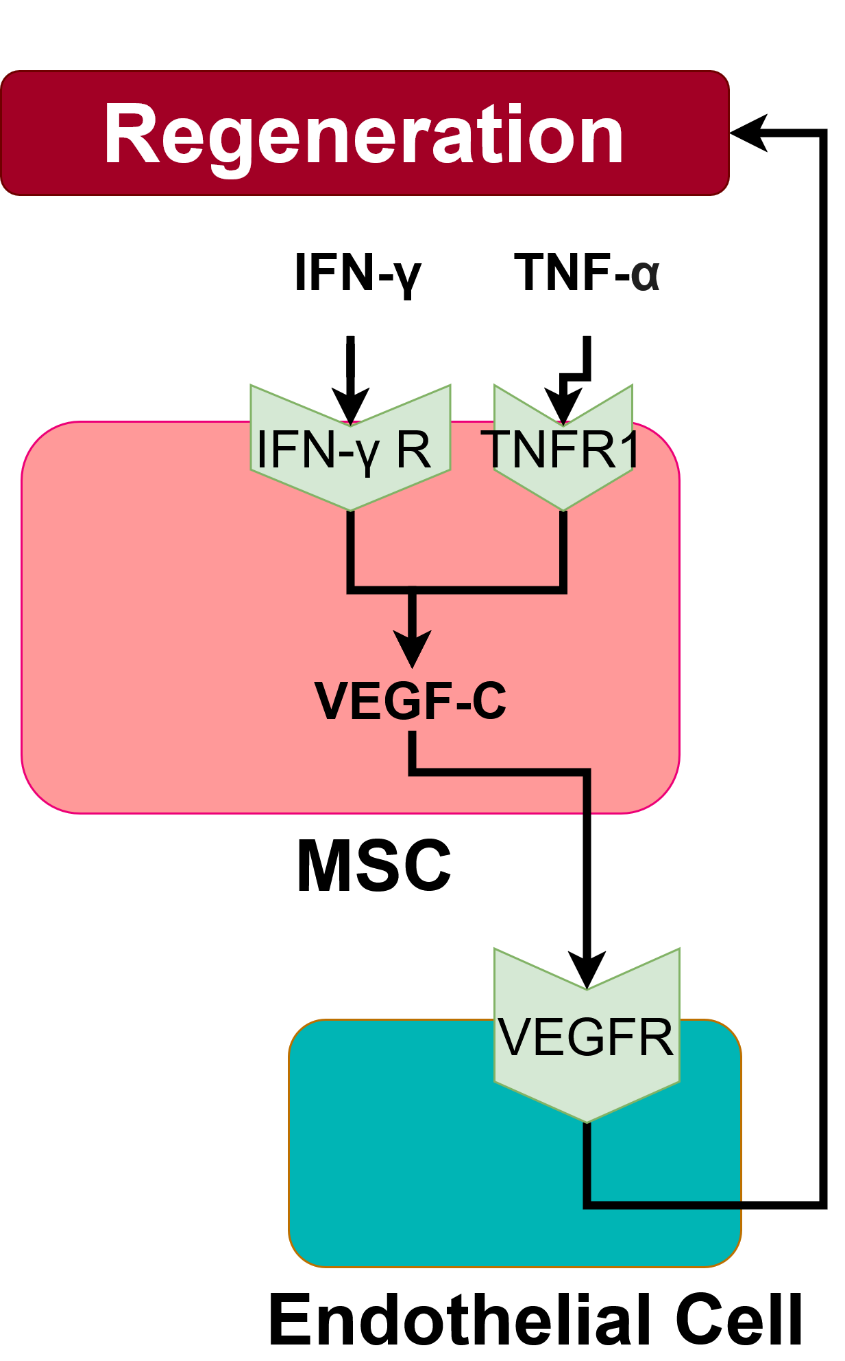


**Supplementary Figure S9: IFN-γ/TNF-α Signaling in Wound Healing.** IFN-γ and TNF-α induce MSCs to secrete more VEGFC. VEGFC primarily binds to VEGFR to promote angiogenesis.

**miR-218 Signaling in Cartilage Regeneration**


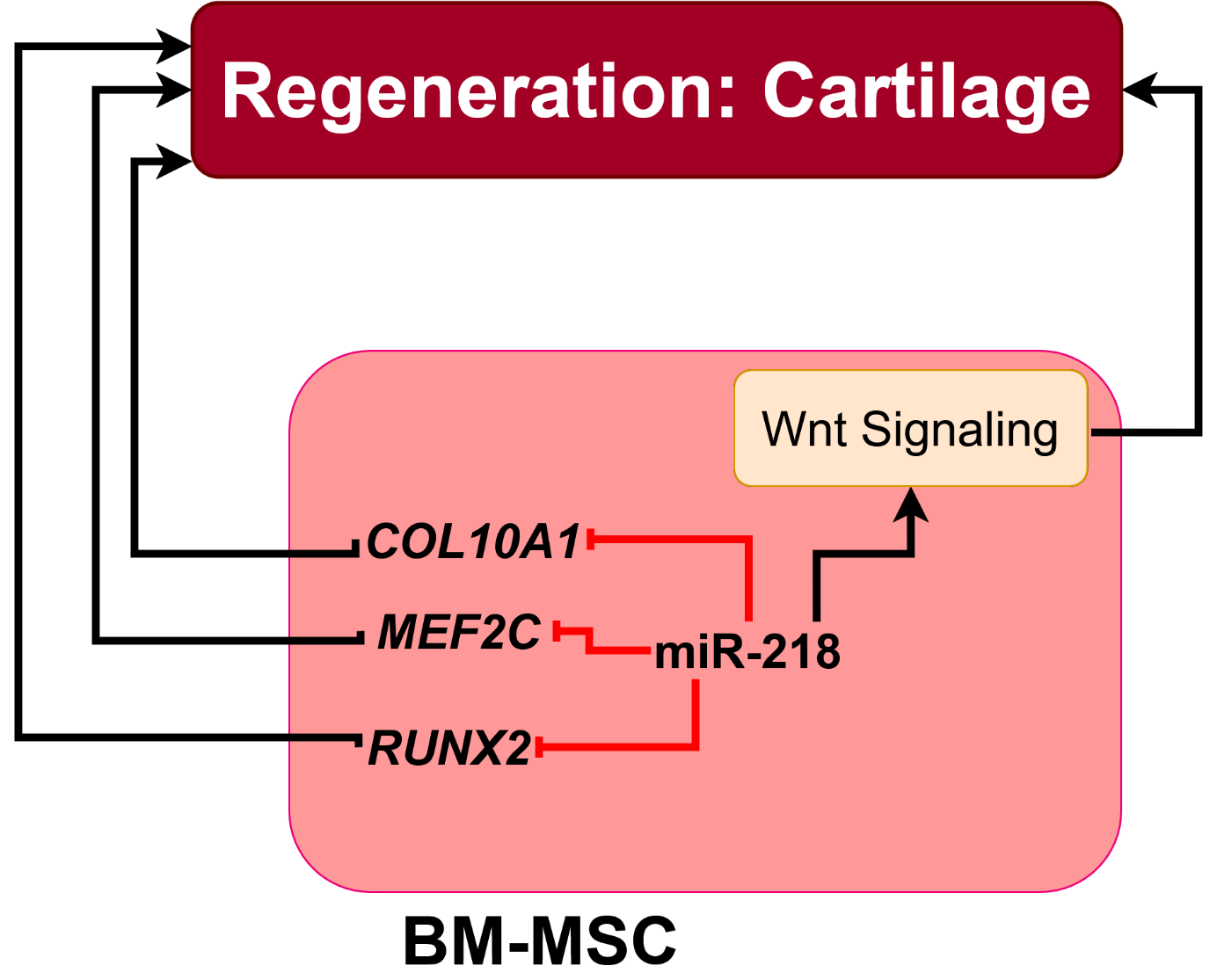
**Supplementary Figure S10: miR-218 Signaling in Cartilage Regeneration.** miR-218 inhibits COL10A1, MEF2C, and RUNX2, the genes involved in chondrogenesis, and induces Wnt signaling, thereby inducing chondrogenesis. This helps to keep in check the regeneration of chondrocytes.

**TGF-β Signaling**


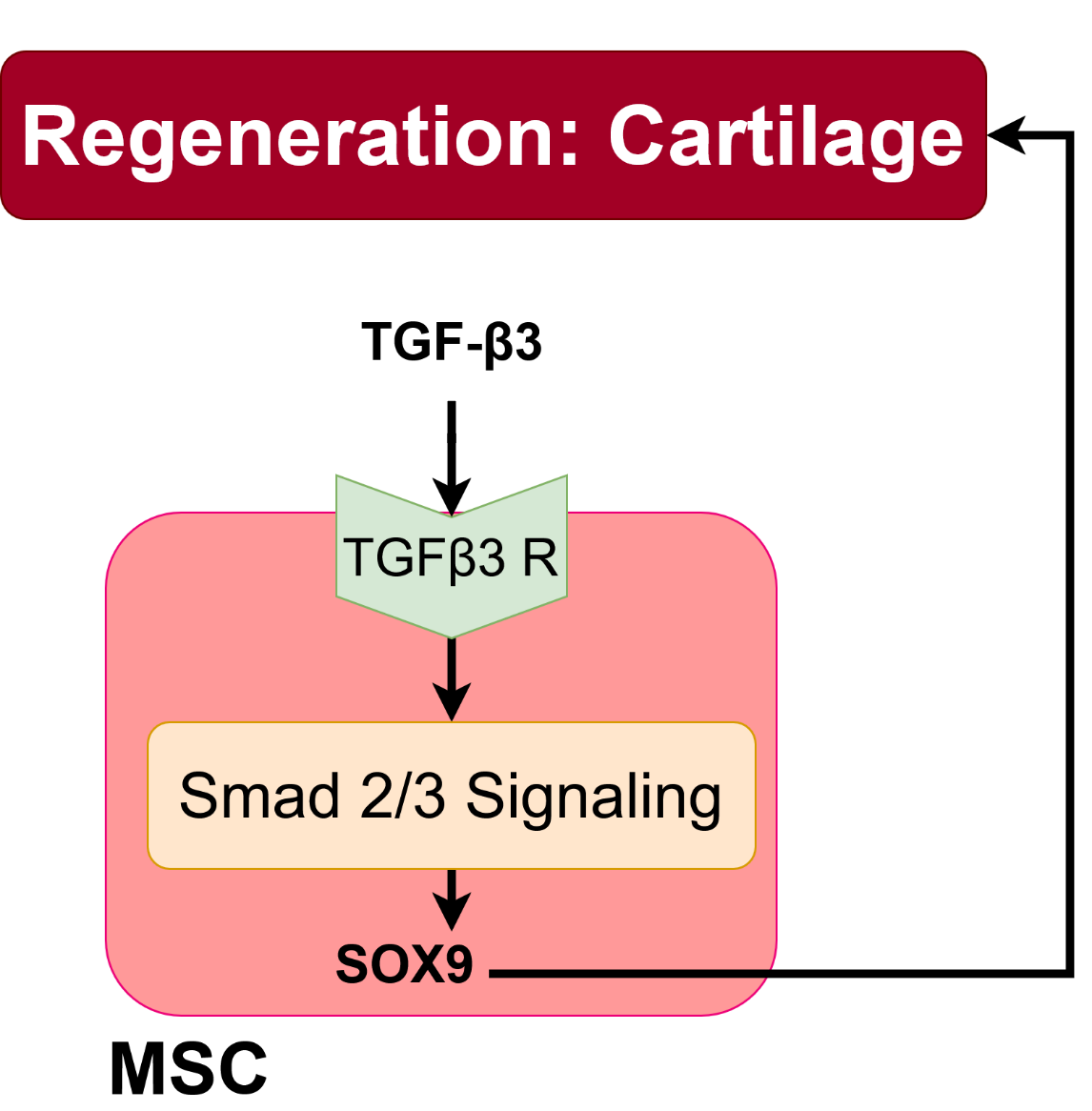


**Supplementary Figure S11: TGF-β Signaling.** TGF-β induces SOX9 expession via Smad2/3 in MSCs leading to cartilage regeneration.


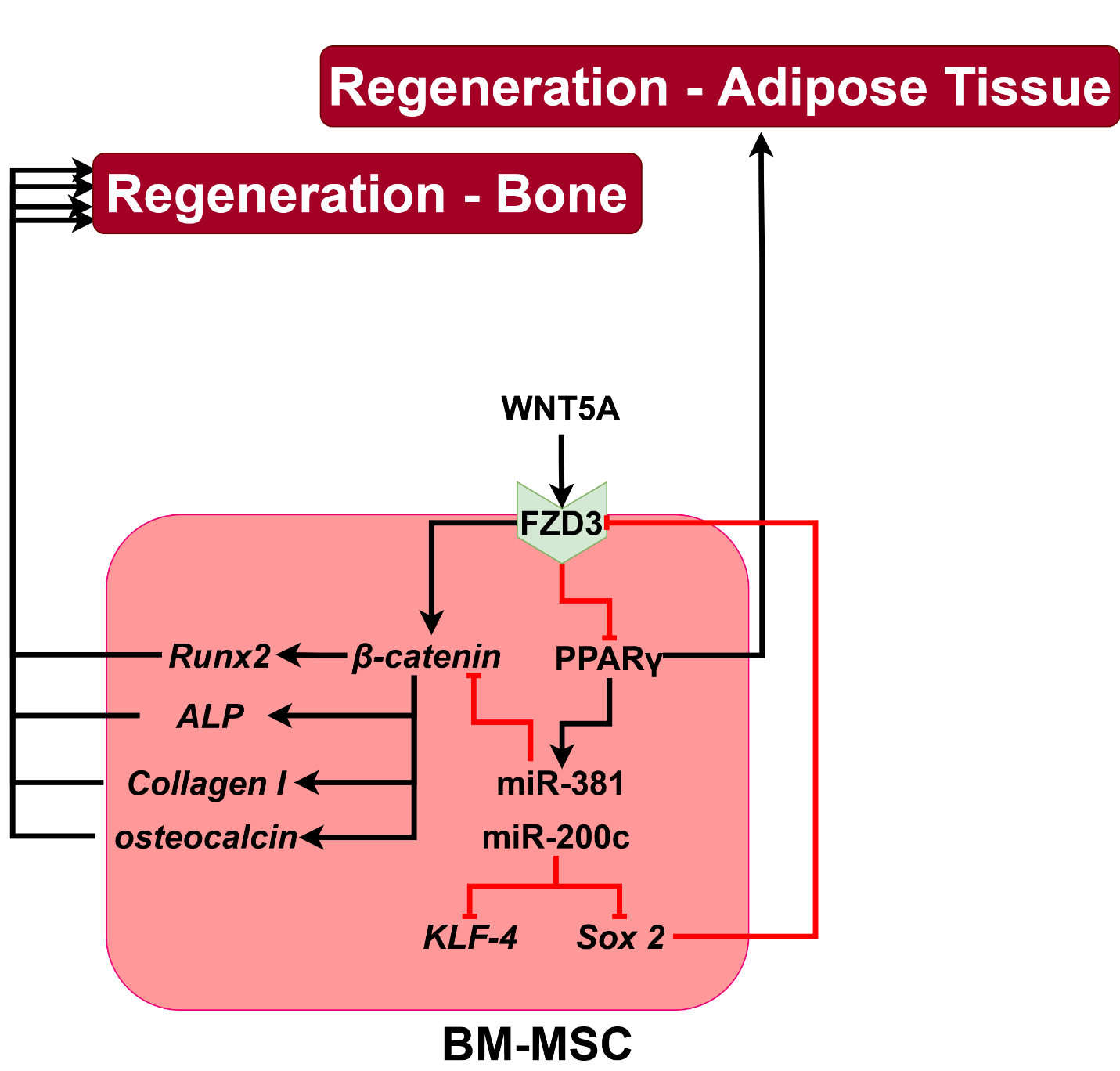
**WNT5A Signaling**

**Supplementary Figure S12: WNT5A Signaling.**  WNT5A binds to its receptor FZD3, thereby induces β-catenin and inhibiting PPARγ, which promotes adipogenesis. β-catenin induces the expression of Runx2, ALP, Collagen I, and osteocalcin, which are important for osteogenesis.

**Retinoic Acid Signaling**


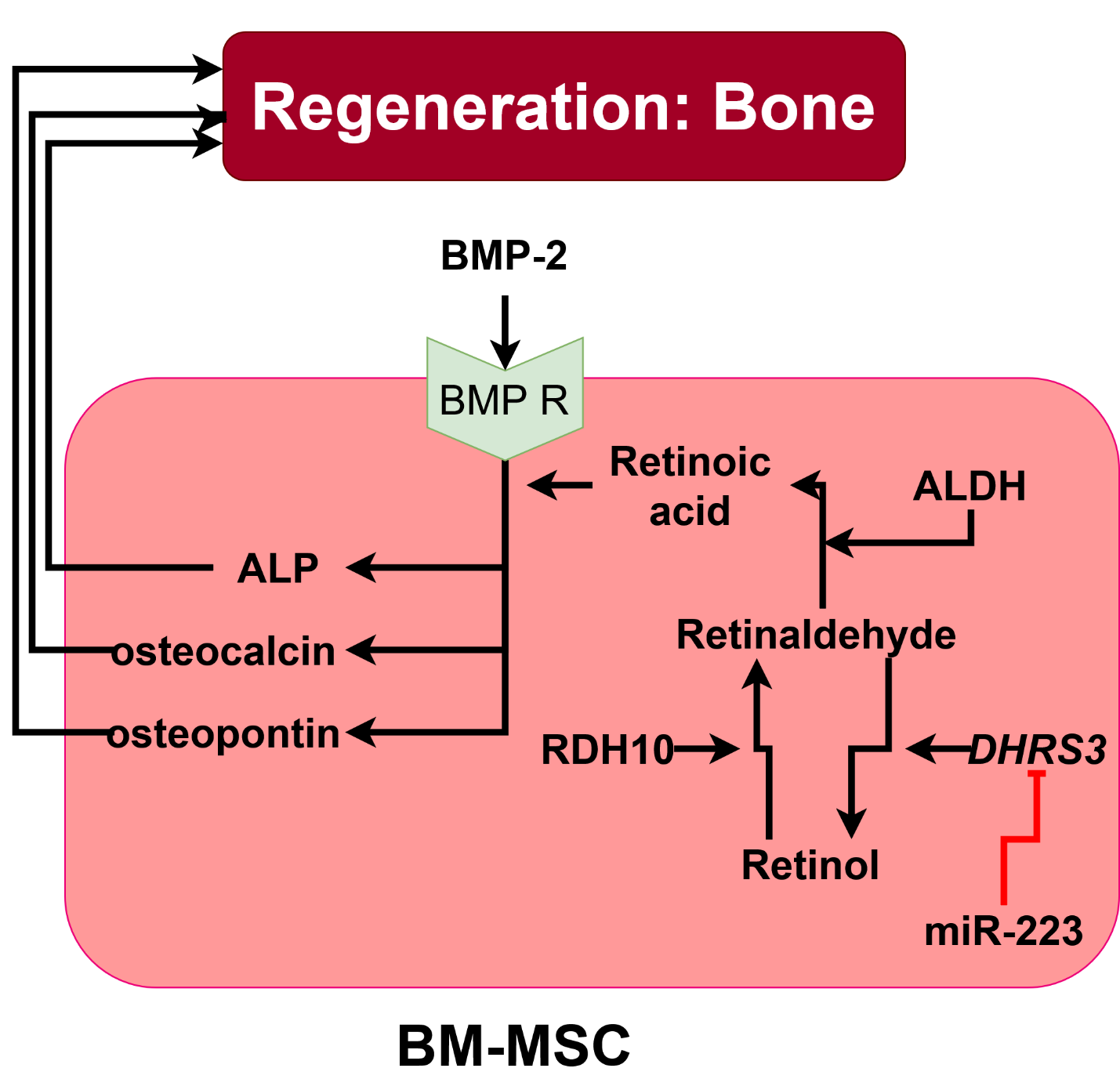


**Supplementary Figure S13:** **Retinoic Acid Signaling**. Retinoic acid is an important factor in BMP induced ALP, osteocalcin, and osteopontin in MSC. miR-223 inhibits DHRS3, that is involved in the production of retinoic acid thereby inhibiting osteoblasts regeneration.

List of Medical Subject Headings (MeSH) keywords to optimize recall and precision of peer-reviewed articles is provided in **Supplementary Table S2**.

**Supplementary Table S2:** MeSH key words used to identify relevant literature.

| MeSH keywords |
| --- |
| mesenchymal stromal cells AND TNF NOT Cancer |
| mesenchymal stromal cells galectin |
| mesenchymal stromal cells AND PGE2 NOT cancer |
| mesenchymal stromal cells AND IL-10 NOT cancer |
| mesenchymal stromal cells AND TSG-6 |
| mesenchymal stromal cells AND Nitric Oxide |
| mesenchymal stromal cells AND TGF-β1 NOT cancer |
| mesenchymal stromal cells AND HGF NOT cancer |
| mesenchymal stromal cells AND UNX2 NOT cancer |
| mesenchymal stromal cells AND TLR2 |
| mesenchymal stromal cells AND TLR4 NOT cancer |
| mesenchymal stromal cells AND TLR3 NOT cancer |
| mesenchymal stromal cells AND Tregs NOT cancer |
| mesenchymal stromal cells AND IFN-γ NOT cancer |
| mesenchymal stromal cells AND IDO NOT cancer |
| mesenchymal stromal cells AND Th17 |
| mesenchymal stromal cells AND IL-17 |
| mesenchymal stromal cells AND Th2 |
| mesenchymal stromal cells AND IL-4 |
| mesenchymal stromal cells AND M2 Macrophages |
| mesenchymal stromal cells AND IL-1β NOT cancer |
| mesenchymal stromal cells AND Wnt NOT cancer |
| mesenchymal stromal cells AND STAT3 |
| mesenchymal stromal cells AND IL-6 NOT cancer |
| mesenchymal stromal cells AND Galectin-3 |
| mesenchymal stromal cells AND VEGF NOT cancer |
| mesenchymal stromal cells AND EGF |
| mesenchymal stromal cells AND IGF-1 |
| mesenchymal stromal cells AND SDF-1 |
| mesenchymal stromal cells AND SCF |
| mesenchymal stromal cells AND angiopoietin 1 |
| mesenchymal stromal cells AND BMP NOT cancer |
| mesenchymal stromal cells AND TNF NOT Cancer |
| mesenchymal stromal cells galectin |
| mesenchymal stromal cells AND PGE2 NOT cancer |
| mesenchymal stromal cells AND IL-10 NOT cancer |
| mesenchymal stromal cells AND TSG-6 |
| mesenchymal stromal cells AND Nitric Oxide |
| mesenchymal stromal cells AND TGF-β1 NOT cancer |
| mesenchymal stromal cells AND HGF NOT cancer |
| mesenchymal stromal cells AND UNX2 NOT cancer |
| mesenchymal stromal cells AND TLR2 |
| mesenchymal stromal cells AND TLR4 NOT cancer |
| mesenchymal stromal cells AND TLR3 NOT cancer |
| mesenchymal stromal cells AND Tregs NOT cancer |
| mesenchymal stromal cells AND IFN-γ NOT cancer |
| mesenchymal stromal cells AND IDO NOT cancer |
| mesenchymal stromal cells AND Th17 |
| mesenchymal stromal cells AND IL-17 |
| mesenchymal stromal cells AND Th2 |
| mesenchymal stromal cells AND IL-4 |
| mesenchymal stromal cells AND M2 Macrophages |
| mesenchymal stromal cells AND IL-1β NOT cancer |
| mesenchymal stromal cells AND Wnt NOT cancer |
| mesenchymal stromal cells AND STAT3 |
| mesenchymal stromal cells AND IL-6 NOT cancer |
| mesenchymal stromal cells AND Galectin-3 |
| mesenchymal stromal cells AND VEGF NOT cancer |
| mesenchymal stromal cells AND EGF |
| mesenchymal stromal cells AND IGF-1 |
| mesenchymal stromal cells AND SDF-1 |
| mesenchymal stromal cells AND SCF |
| mesenchymal stromal cells AND angiopoietin 1 |
| mesenchymal stromal cells AND BMP NOT cancer |

**Supplementary Table S3:** Surface markers of unstimulated MSCs based on their source

| **Type of MSC** | **Surface Markers Positive** | **Surface Markers Negative** | **References** |
| --- | --- | --- | --- |
| Gingivial MSC | CD73, CD90, CD105, SSEA-4, STRO-1 | CD34 and CD45 (hematopoietic cell markers) | (25) |
| Menstrual Blood-Derived MSCs | CD9, CD29, CD44, CD73, CD90, CD105, CD166, HLA-A, HLA-B, HLA-C, and OCT-4, | CD19, CD 34, CD45, CD133, and HLA-DR; | (26,27) |
| Wharton's Jelly MSCs | CD44, CD90, CD105 | CD34 and CD45 | (28,29) |
| Tonsil MSC | CD73, CD90, CD105, CD29, CD44, CD166, CD58, and CD49e. | CD14, CD34, CD45, and CD133 (hematopoietic markers), CD31 (endothelial marker), and CD40, CD80, and CD86 (co-stimulatory proteins), class II MHC antigens are entirely absent on T-SCs | (30,31) |
| UC-MSC | HLA-ABC, CD29, CD44, CD73, CD90, and CD105 | HLA-DR, CD34, and CD45 | (32,33) |
| AM-MSC | CD29, CD44, CD73, CD90, CD10, CD14, CD19, CD3^-^, CD45, and HLA-DR | CD14-PE and CD45-FITC | (34–36) |
| AD-MSC | CD9, CD10, CD13, CD29, CD44, CD49d (integrin α4), CD49e, CD54, CD55, CD73, CD90, CD105, CD106, CD146, CD166, and STRO-1 (stromal precursor antigen-1), | CD 14, CD19 (B4), CD34 (Mucosialin), CD45, CD16 (FcγRIII), CD56, CD61 (integrin β3), CD62E (E-selectin), CD104 (integrin β4), and CD106, CD31 and CD144 | (37,38) |
| BM-MSC | CD105+, CD73+ and CD90+, CD44+, CD166+, CD29+, STRO-1+, CD146+, and CD271+ | CD45, CD34, CD14, CD11b, CD79a, CD19 and HLA-DR. | (39,40) |

MSC – mesenchymal stromal cells, CD – cluster of differentiation, SSEA-4 – stage-specific embryonic antigen-4, STRO-1 – mesenchyme 1, HLA – human leukocyte antigen, OCT-4 – octamer-binding transcription factor 4, MHC – major histocompatibility complex, UC-MSC - umbilical cord-derived mesenchymal stromal cells, AM-MSC – amniotic membrane mesenchymal stromal cells, AD-MSC – adipose-derived mesenchymal stromal cells, BM-MSC – bone marrow mesenchymal stromal cells.

**Supplementary References:**

1. Al-Lazikani B, Banerji U, Workman P. Combinatorial drug therapy for cancer in the post-genomic era. *Nat Biotechnol* (2012) 30:679–92. doi: 10.1038/nbt.2284

2. Ayyadurai VAS, Dewey CF. CytoSolve: A scalable computational method for dynamic integration of multiple molecular pathway models. *Cell Mol Bioeng* (2011) 4:28–45. doi: 10.1007/s12195-010-0143-x

3. Koo A, Nordsletten D, Umeton R, Yankama B, Ayyadurai S, García-Cardeña G, Dewey CF. In Silico Modeling of Shear-Stress-Induced Nitric Oxide Production in Endothelial Cells through Systems Biology. *Biophys J* (2013) 104:2295–2306. doi: 10.1016/j.bpj.2013.03.052

4. Nordsletten DA, Yankama B, Umeton R, Ayyadurai VAS, Dewey CF. Multiscale mathematical modeling to support drug development. *IEEE Trans Biomed Eng* (2011) doi: 10.1109/TBME.2011.2173245

5. Sweeney MD, Ayyadurai S, Zlokovic B V. Pericytes of the neurovascular unit: Key functions and signaling pathways. *Nat Neurosci* (2016) 19:771–783. doi: 10.1038/nn.4288

6. Ayyadurai VAS, Deonikar P, Ali A, Rockel J, Kapoor M. Molecular Systems Architecture of Human Knee Osteoarthritis. *CytoSolve, Inc* (2020)

7. Ayyadurai VAS, Deonikar P, McLure KG, Sakamoto KM. Molecular Systems Architecture of Interactome in the Acute Myeloid Leukemia Microenvironment [Supplemental Material]. *Cancers (Basel)* (2022) 14: doi: 10.3390/CANCERS14030756

8. Al-Lazikani B, Banerji U, Workman P. Combinatorial drug therapy for cancer in the post-genomic era. *Nat Biotechnol* (2012) 30:679–92. doi: 10.1038/nbt.2284

9. Ayyadurai VAS, Deonikar P. Do GMOs Accumulate Formaldehyde and Disrupt Molecular Systems Equilibria? Systems Biology May Provide Answers. *Agricultural Sciences* (2015) 06:630–662. doi: 10.4236/as.2015.67062

10. Mohan M, Kothandaram S, Venugopal V, Deonikar P, Ayyadurai VAS. Integrative Modeling of Oxidative Stress and C1 Metabolism Reveals Upregulation of Formaldehyde and Downregulation of Glutathione. *Am J Plant Sci* (2015) 06:1527–1542. doi: 10.4236/ajps.2015.69152

11. Shiva Ayyadurai VA, Hansen M, Fagan J, Deonikar P. &lt;i&gt;In-Silico&lt;/i&gt; Analysis &amp; &lt;i&gt;In-Vivo&lt;/i&gt; Results Concur on Glutathione Depletion in Glyphosate Resistant GMO Soy, Advancing a Systems Biology Framework for Safety Assessment of GMOs. *Am J Plant Sci* (2016) 07:1571–1589. doi: 10.4236/ajps.2016.712149

12. Kothandaram S, Deonikar P, Mohan M, Venugopal V, Ayyadurai VAS. &lt;i&gt;In Silico&lt;/i&gt; Modeling of C1 Metabolism. *Am J Plant Sci* (2015) 06:1444–1465. doi: 10.4236/ajps.2015.69144

13. Ayyadurai VAS, Deonikar P. Bioactive compounds in green tea may improve transplant tolerance: A computational systems biology analysis. *Clin Nutr ESPEN* (2021) 46:439–452. doi: 10.1016/j.clnesp.2021.09.012

14. Ayyadurai VAS, Deonikar P, Bannuru RR. Attenuation of low-grade chronic inflammation by phytonutrients: A computational systems biology analysis. *Clin Nutr ESPEN* (2022) doi: 10.1016/J.CLNESP.2022.03.010

15. Cornish-Bowden A. One hundred years of Michaelis–Menten kinetics. *Perspect Sci (Neth)* (2015) doi: 10.1016/j.pisc.2014.12.002

16. Michaelis VL, Maud Menten ML, Goody RS, Johnson KA. (1890) 57, 834. 6 A. J. Brown. *J Chem Soc* (1902)

17. Hucka M, Finney A, Sauro HM, Bolouri H, Doyle JC, Kitano H, Arkin AP, Bornstein BJ, Bray D, Cornish-Bowden A, et al. The systems biology markup language (SBML): A medium for representation and exchange of biochemical network models. *Bioinformatics* (2003) 19:524–531. doi: 10.1093/bioinformatics/btg015

18. Ayyadurai VAS. “Services-Based Systems Architecture for Modeling the Whole Cell: A Distributed Collaborative Engineering Systems Approach.,” (2010). p. 115–168 doi: 10.1007/8754_2010_1

19. Ayyadurai VAS. “Services-Based Systems Architecture for Modeling the Whole Cell: A Distributed Collaborative Engineering Systems Approach.,” (2010). p. 115–168 doi: 10.1007/8754_2010_1

20. Oden JT (John T, Reddy JN (Junuthula N. *An Introduction to the Mathematical Theory of Finite Elements*. 1976th ed. New York: Dover Publications. (2011). 429 p.

21. Shmulevich I, Aitchison JD. DETERMINISTIC AND STOCHASTIC MODELS OF GENETIC REGULATORY NETWORKS. *Methods Enzymol* (2009) 467:335. doi: 10.1016/S0076-6879(09)67013-0

22. Cumming G, Fidler F, Vaux DL. Error bars in experimental biology. *J Cell Biol* (2007) 177:7. doi: 10.1083/JCB.200611141

23. Thanh VH, Zunino R, Priami C. Efficient finite-difference method for computing sensitivities of biochemical reactions. *Proceedings of the Royal Society A: Mathematical, Physical and Engineering Sciences* (2018) 474: doi: 10.1098/rspa.2018.0303

24. Thanh VH, Zunino R, Priami C. Efficient finite-difference method for computing sensitivities of biochemical reactions. *Proceedings of the Royal Society A: Mathematical, Physical and Engineering Sciences* (2018) 474: doi: 10.1098/rspa.2018.0303

25. Peng Y, Jaar J, Tran SD. Gingival mesenchymal stem cells: Biological properties and therapeutic applications. *J Oral Biol Craniofac Res* (2024) 14:547–569. doi: 10.1016/j.jobcr.2024.07.003

26. Chen L, Qu J, Cheng T, Chen X, Xiang C. Menstrual blood-derived stem cells: toward therapeutic mechanisms, novel strategies, and future perspectives in the treatment of diseases. *Stem Cell Res Ther* (2019) 10:406. doi: 10.1186/s13287-019-1503-7

27. Wu Q, Wang Q, Li Z, Li X, Zang J, Wang Z, Xu C, Gong Y, Cheng J, Li H, et al. Human menstrual blood-derived stem cells promote functional recovery in a rat spinal cord hemisection model. *Cell Death Dis* (2018) 9:882. doi: 10.1038/s41419-018-0847-8

28. Hu Y, Liang J, Cui HP, Wang XM, Rong H, Shao B, Cui H. Wharton’s jelly mesenchymal stem cells differentiate into retinal progenitor cells. *Neural Regen Res* (2013) 8:1783–1792. doi: 10.3969/j.issn.1673-5374.2013.19.006

29. Abouelnaga H, El-Khateeb D, Moemen Y, El-Fert A, Elgazzar M, Khalil A. Characterization of mesenchymal stem cells isolated from Wharton’s jelly of the human umbilical cord. *Egyptian Liver Journal* (2022) 12:2. doi: 10.1186/s43066-021-00165-w

30. Oh S-Y, Choi YM, Kim HY, Park YS, Jung S-C, Park J-W, Woo S-Y, Ryu K-H, Kim HS, Jo I. Application of Tonsil-Derived Mesenchymal Stem Cells in Tissue Regeneration:  Concise Review. *Stem Cells* (2019) 37:1252–1260. doi: 10.1002/stem.3058

31. Cho K-A, Lee HJ, Jeong H, Kim M, Jung SY, Park HS, Ryu K-H, Lee SJ, Jeong B, Lee H, et al. Tonsil-derived stem cells as a new source of adult stem cells. *World J Stem Cells* (2019) 11:506–518. doi: 10.4252/wjsc.v11.i8.506

32. Chang YH, Ding DC, Wu KC. Human Umbilical Mesenchymal Stromal Cells Mixed with Hyaluronan Transplantation Decreased Cartilage Destruction in a Rabbit Osteoarthritis Model. *Stem Cells Int* (2021) 2021: doi: 10.1155/2021/2989054

33. Chang Y-H, Wu K-C, Liu H-W, Chu T-Y, Ding D-C. Human umbilical cord-derived mesenchymal stem cells reduce monosodium  iodoacetate-induced apoptosis in cartilage. *Tzu Chi Med J* (2018) 30:71–80. doi: 10.4103/tcmj.tcmj_23_18

34. Teoh PL, Mohd Akhir H, Abdul Ajak W, Hiew VV. Human Mesenchymal Stromal Cells Derived from Perinatal Tissues: Sources,  Characteristics and Isolation Methods. *Malays J Med Sci* (2023) 30:55–68. doi: 10.21315/mjms2023.30.2.5

35. Lin F-H, Yang Y-X, Wang Y-J, Subbiah SK, Wu X-Y. Amniotic membrane mesenchymal stromal cell-derived secretome in the treatment of  acute ischemic stroke: A case report. *World J Clin Cases* (2023) 11:6543–6550. doi: 10.12998/wjcc.v11.i27.6543

36. Dizaji Asl K, Shafaei H, Soleimani Rad J, Nozad HO. Comparison of Characteristics of Human Amniotic Membrane and Human Adipose Tissue  Derived Mesenchymal Stem Cells. *World J Plast Surg* (2017) 6:33–39.

37. Huang S-J, Fu R-H, Shyu W-C, Liu S-P, Jong G-P, Chiu Y-W, Wu H-S, Tsou Y-A, Cheng C-W, Lin S-Z. Adipose-Derived Stem Cells: Isolation, Characterization, and Differentiation Potential. *Cell Transplant* (2013) 22:701–709. doi: 10.3727/096368912X655127

38. Krawczenko A, Klimczak A. Adipose Tissue-Derived Mesenchymal Stem/Stromal Cells and Their Contribution to  Angiogenic Processes in Tissue Regeneration. *Int J Mol Sci* (2022) 23: doi: 10.3390/ijms23052425

39. Fonseca LN, Bolívar-Moná S, Agudelo T, Beltrán LD, Camargo D, Correa N, Del Castillo MA, Fernández de Castro S, Fula V, García G, et al. Cell surface markers for mesenchymal stem cells related to the skeletal system: A scoping review. *Heliyon* (2023) 9:e13464. doi: https://doi.org/10.1016/j.heliyon.2023.e13464

40. Kuchakzadeh F, Ai J, Ebrahimi-Barough S. Tissue engineering and stem cell-based therapeutic strategies for premature ovarian insufficiency. *Regen Ther* (2024) 25:10–23. doi: https://doi.org/10.1016/j.reth.2023.11.007
